# Lusca: FIJI (ImageJ) based tool for automated morphological analysis of cellular and subcellular structures

**DOI:** 10.1038/s41598-024-57650-6

**Published:** 2024-03-28

**Authors:** Iva Šimunić, Denis Jagečić, Jasmina Isaković, Marina Dobrivojević Radmilović, Dinko Mitrečić

**Affiliations:** 1 Department of Histology and Embryology, University of Zagreb School of Medicine, 10000 Zagreb, Croatia; 2 Laboratory for Stem Cells, Department for Regenerative Neuroscience, Croatian Institute for Brain Research, University of Zagreb School of Medicine, 10000 Zagreb, Croatia; 3 School of Medicine, European University Cyprus – Frankfurt Branch, 60488 Frankfurt am Main, Germany; 4 Laboratory for Regenerative Neuroscience, Department for Regenerative Neuroscience, Croatian Institute for Brain Research, University of Zagreb School of Medicine, 10000 Zagreb, Croatia

**Keywords:** Biological techniques, Computational biology and bioinformatics, Neuroscience, Stem cells

## Abstract

The human body consists of diverse subcellular, cellular and supracellular structures. Neurons possess varying-sized projections that interact with different cellular structures leading to the development of highly complex morphologies. Aiming to enhance image analysis of complex biological forms including neurons using available FIJI (ImageJ) plugins, Lusca, an advanced open-source tool, was developed. Lusca utilizes machine learning for image segmentation with intensity and size thresholds. It performs particle analysis to ascertain parameters such as area/volume, quantity, and intensity, in addition to skeletonization for determining length, branching, and width. Moreover, in conjunction with colocalization measurements, it provides an extensive set of 29 morphometric parameters for both 2D and 3D analysis. This is a significant enhancement compared to other scripts that offer only 5–15 parameters. Consequently, it ensures quicker and more precise quantification by effectively eliminating noise and discerning subtle details. With three times larger execution speed, fewer false positive and negative results, and the capacity to measure various parameters, Lusca surpasses other existing open-source solutions. Its implementation of machine learning-based segmentation facilitates versatile applications for different cell types and biological structures, including mitochondria, fibres, and vessels. Lusca’s automated and precise measurement capability makes it an ideal choice for diverse biological image analyses.

## Introduction

The human body is composed of diverse cell types and structures with various shapes. Neurons, for instance, possess projections, axons and dendrites, that vary in size and intermingle with other cellular structures to form very complex morphologies. In addition, such projections, and their endings, crucial for cellular communication, are not static. Rather, they dynamically adjust their morphology in response to the physiological and pathological changes within tissue. Moreover, even the subcellular structures, like mitochondria, or supracellular, like vessels, adapt their shapes based on the cellular or tissue environment.

Advanced cell and tissue visualization methods generate a vast number of images filled with numerous complex cellular and subcellular structures. Algorithms that automatically recognize, separate, and quantify these elements are crucial for detailed analysis of such images as manual measurements, though meticulous, are time-consuming, thereby often limiting analyses to just a fraction of cell populations^1^. Nowadays, specialized platforms as Imaris or MATLAB offer analysis options, but they require a substantial budget. Although powerful tools, Imaris might still require some manual interventions during the analysis, while MATLAB’s 3DMorph script, developed primarily for the analysis of 3D microglial morphology, can yield false positive or false negative results when analysing neurons, since it includes neuronal bodies in length measurements^2^. On the other hand, open-source platforms like ImageJ and Cell Profiler lack specialization for detailed analyses of elongated shapes of biological structures^3,4^. Therefore, plugins and macros are created to surpass manual analysis, accelerating and optimizing image analysis in open-source software. These scripts, however, often struggle with accurate recognition of neural bodies and projections, particularly in low-contrast or noisy images, do not support 3D analyses, and demand manual steps that prolong analysis time.

Analysing neural cell projections commonly involves laborious manual or semi-manual methods in programs like NeuronJ^5^. However, this process is time-consuming and biased due to human subjectivity and is, therefore, typically used for tracing only a few sections of neurons. Other programs like NeuroMantic, NeuronStudio, and Vaa3D use semi-automatic reconstruction for faster analysis, but the emergence of fully automated algorithms has notably accelerated the image analysis process^6–9^. However, fully automated ImageJ/FIJI algorithms like Neurite Tracer, NeurphologyJ, and AutoNeuriteJ, lack certain valuable features. Namely, they offer automated analysis of only a limited number of neural morphological parameters, lack 3D analysis capabilities, and ignore objects partially exiting the field of view, limiting analysis of more mature neuron cultures^10–12^.

Considering the aforementioned limitations, the main goal of this study was to create a fast and high-throughput algorithm to analyse neuronal morphology, Lusca (iLUminating SCience Algoritm), using the open-source software FIJI (ImageJ) along with its plugins. Lusca automates and accelerates analyses of neural images, providing an objective, accurate, and time-efficient alternative to current open-source tools. This macro can be used to quantify the length, width, number, branching, intensity, volume, area, and colocalization on both 2D and 3D images, effectively distinguishing relevant structures from background and noise artifacts. Furthermore, it facilitates processing of results obtained with various imaging methods like microscopy and MR imaging, successfully analysing diverse subcellular, cellular, and supracellular structures such as projections, mitochondria, lysosomes, and vessels.

## Results

### Description of the Lusca algorithm

The image analysis by Lusca includes the following steps: (1) setting of the input parameters, (2) segmenting and precise tuning of the image to obtain the masked region of interest (ROI), and (3) quantifying recognized objects via particle, skeleton, and colocalization analyses.

### Setting up the input parameters for image analysis

Lusca’s wizard guides users through the selection of input parameters: (a) choosing the image folder, (b) selecting the image type (e.g., channel/single, 2D/3D), and (c) optionally setting scale, cropping the image for analysis and/or the user can proceed to quantify other morphological parameters with interactive approach, without interactive approach or with already segmented images. For further analysis without an interactive approach, the user provides (d) the location of the folder containing the classifier(s), (e) the name of the image segmentation classifier, (f) the intensity, area/volume, and circularity thresholds for fine-tuning, and selects (g) the type of morphological analysis (*neural projections, soma and nuclei, area, number and intensity, length and branching, width, and colocalization of segments*). Additional input settings for specific analyses include (h) histogram parameters (number of bins, minimum and maximum width) for *neural projections* or *width analysis*, (i) parameters from (d) to (f) corresponding to nuclei image analysis for *soma analysis*, and (j) parameters from (d) to (f) corresponding to colocalizing image analysis for *colocalization of segments*.

**Figure 1:**
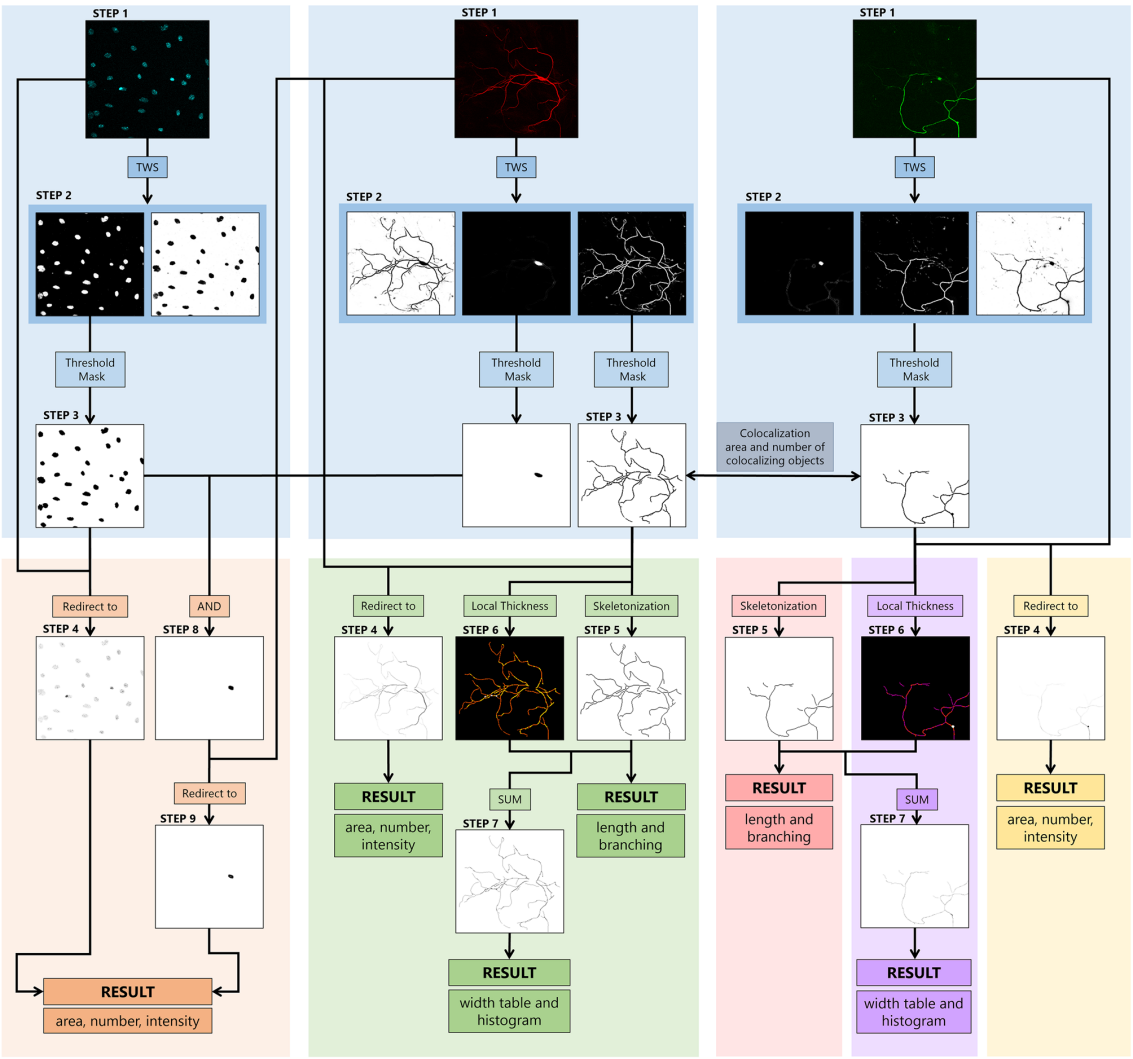
Pipeline for image analysis of neural projections and bodies. Blue squares represent image segmentation, while grey, orange, green, red, violet, and yellow represent different options for image analysis. These include colocalization, neural bodies, neural projections, length and branching, width, and area, number, and intensity, respectively. Step 1 shows the enlarged input images of neurons and nuclei stained with MAP2 (red), SMI312 (green), and DAPI (cyan). ROIs are segmented from the background with TWS in step 2, following intensity, area, and circularity thresholding to obtain the segmented image in step 3. “Neural bodies” analysis includes the calculation of area, number, and intensity for both nuclei and soma images. Neural bodies image is acquired by using the Boolean operator “AND” on previously dilated nuclei image and neuron image from step 3 to avoid false signals. The area, number, and intensity of both neural bodies and nuclei are calculated with the particle analyser after redirection to the corresponding input images (orange square, step 4). Analysis option “Neural projections” includes the calculation of area, number, intensity, length, branching, and width. Particle analysis was performed again to obtain area, number, and intensity results (green square, step 4). Length and branching are calculated after forming skeletons in step 5, while width calculation further involves the transformation of the step 5 image into a 32-bit image. Following thresholding and deduction of 255, values of NaN for the background pixels, and 0 for skeletons, are obtained. Simultaneously, using the Local Thickness on the image from step 3, accurate width dimensions are achieved (step 6). The images from step 5 and step 6 are added to obtain the image in step 7 from which width results are calculated. The separation of these options into distinct squares (red, violet, and yellow) facilitates better user comprehension and easier application, especially considering the versatility of Lusca in analysing various biological objects beyond neurons.

If the user chooses the interactive approach for quantification of other morphological parameters, the wizard guides them through the image analysis process to create and set unknown variables from (d) to (j). A sequence of “while” loops is used to repeat the steps until the user is satisfied with the obtained image. A detailed breakdown of the aforementioned macro’s architecture can be found in Fig. S1. On the other hand, if morphological analysis with already segmented images is chosen, the user only provides the location of those segmented images, so the analysis starts from step 3 in the Fig. 1.

By the end of this step, the images from the folder are saved in the LIST data type, while the input data for each image type is saved into arrays. This approach allows for automated loading of images and provides the user with a sorted array of a vast amount of data for image analysis. Additionally, a "Results" folder is created within the image directory for users to access images, graphs, and tables displaying the analysis results.

### Image segmentation and fine-tuning

For image segmentation, Lusca relies on Trainable Weka Segmentation (TWS), a machine-learning plugin^13^. This process involves forming a machine-learning classifier by defining classes that represent the number of objects the user wishes to distinguish within the image. Initially, a minimum of two classes is required, with the option to add more classes through “Create new class”, focusing on the shared intensity and shape characteristics. Once a classifier of satisfactory precision is created, it should be saved using the “Save classifier” command, enabling its limitless utilization in automated image processing of similar types of data. For quantification purposes, used classifiers were random forest models. Therefore, since the decision tree structure is determined by the input pixels, precise determination of ROIs is crucial. Furthermore, the selection of suitable training features, which rely on the characteristics of the object intended for segmentation, is equally important. These affect the learning process, optimizing both image segmentation and analysis speed.

TWS as output generates Probability Maps, a stack with channels matching the segmentation classes. For instance, in Fig. 1 step 1, an image of neurons was segmented into projections, somas, and the background (step 2). In each channel, the whiter the pixels are, the greater the possibility that a certain pixel belongs to the selected class. Additional fine-tuning is performed by applying intensity, area/volume, and circularity thresholds to obtain a segmented image, represented by black masks on a white background (Fig. 1, step 3).

### Image quantification procedure

To analyse segmented objects, the segmented image is redirected to the input image, replacing black masks with shaded masks based on the intensity of the input image (Fig. 1, step 4). The Particle Analyzer measures 2D image parameters like number, area, circularity, and intensity, while the 3D Objects Counter quantifies number, volume, surface, sphericity, and intensity for 3D images^14,15^.

Although designed for analysis of neuronal projections, Lusca can also analyse neuronal bodies (Fig. 1, orange square). During the initial testing, the image segmentation pipeline had inaccuracies when analysing images with thicker neurites, increased background noise, and stain accumulation, which were mistaken for neuron bodies. This happened since TWS, and particle analyser could not eliminate these false positive signals due to their similarity in characteristics, size, or shape to actual neuron bodies. Since nuclei reside within the cell bodies, and seldom intersect with the aforementioned inaccuracies, extraction of the overlapping signal allowed for the separation of neuronal bodies. Considering the nuclei are smaller than somas, the nuclei's surface area was enlarged before applying the “AND” operator (Fig. 1, steps 8–9). This ensured accurate cell body recognition, facilitating calculations of area/volume, surface, number, intensity, and shape.

To quantify the length of projections, the objects from step 3, derived from the segmented image, are converted into one-pixel-width lines, i.e. skeletons, using the Skeletonize (2D/3D) plugin (Fig. 1, step 5)^16^. Following skeleton analysis yields data on endpoint, junction, and slab voxels, as well as the maximum, mean, and sum of branch lengths, offering insights into the neuronal culture's condition.

The width of the projections is determined by calculating the width per each increment of length. This is achieved by applying a Local Thickness plugin on the image from step 3^17^. The width corresponds to the largest circle (2D) or sphere (3D) that fits within the projection. Simultaneously, the skeleton image (step 5) becomes a 32-bit image, so that after thresholding background's value is converted to Not a Number (NaN), while skeleton pixels remain at a value of 255. To establish a direct connection between thickness in step 6, and the corresponding pixel in step 5, 255 is subtracted from the whole image leaving the background pixels at NaN, and skeleton pixels at 0. Merging these images creates a final image where pixel values directly indicate section thickness (step 7). From this image histogram, mean, and median width are calculated.

Colocalization analysis is performed on segmented objects within the selected images (Fig. 1, step 3). Manders’ Colocalization Coefficient is determined as a quotient of colocalized area/volume between the two images and the total area/volume on one of the images^18^. To obtain the pixels with positive signals on both images, Lusca uses the Boolean operator “AND” with the particle analyser for area measurements, while 3D objects counter is utilized for volume measurements. The total area/volume of the image objects is acquired after segmentation, as mentioned earlier in this paragraph, with the particle analyser or 3D objects counter.

### Validation of Lusca and comparison to other algorithms

To validate the results obtained with Lusca, the manual analysis served as the golden standard.

The comparison of Lusca’s results primarily centred on open-source scripts such as NeuronJ, NeuriteTracer, NeurphologyJ, and CellProfiler.

The comparison criteria included parameters readily accessible in these programs, excluding those requiring additional user input. Key parameters defining Lusca’s performance were established: projection length and width, soma and nuclei counts, neuron and nuclei area/volume, and analysis time.

Additionally, an execution speed parameter was introduced, calculated as the quotient of the number of measurements and analysis time using PowerShell. This facilitated a comparative analysis of the efficiency and speed of the different programs in executing varied functions within a defined time frame.

Thirty images of neurons and nuclei were used for validation and comparison, categorized into three groups: 2D high-quality-stained, 2D low-quality-stained, and 3D high-quality-stained images.

High-quality-stained images exhibited minimal noise between neuronal or nuclear areas and their boundaries, with a ratio between area intensity and boundary intensity greater than 5. On the other hand, low-quality-stained images had a high amount of background noise, so the aforementioned ratio was lower than 5^19^. To assess the program performance across diverse cell cultures, capturing various morphological features, neurons were cultured for 5–10 days.

### Measurement of neuronal projections reveals that Lusca achieves the same level of preciseness as manual analyses with 230 times larger execution speed

To evaluate the accuracy, precision, and speed of Lusca, it was first compared to the “golden standard”—manual analyses. A detailed breakdown of manual validation can be found in the Methods section. No statistically significant differences were found between manual analyses and Lusca for both 2D high and low-quality images, along with 3D images for neuronal body and nuclei counts, neuron and nuclei area/volume, as well as projection length and width. A detailed description of data, shown as mean and standard deviation (SD), can be found in Supplements (Tables S1–S3).

### Comparison of Lusca with other image analysis algorithms reveals that Lusca offers the largest number of measurements with the highest execution speed

**Figure 2:**
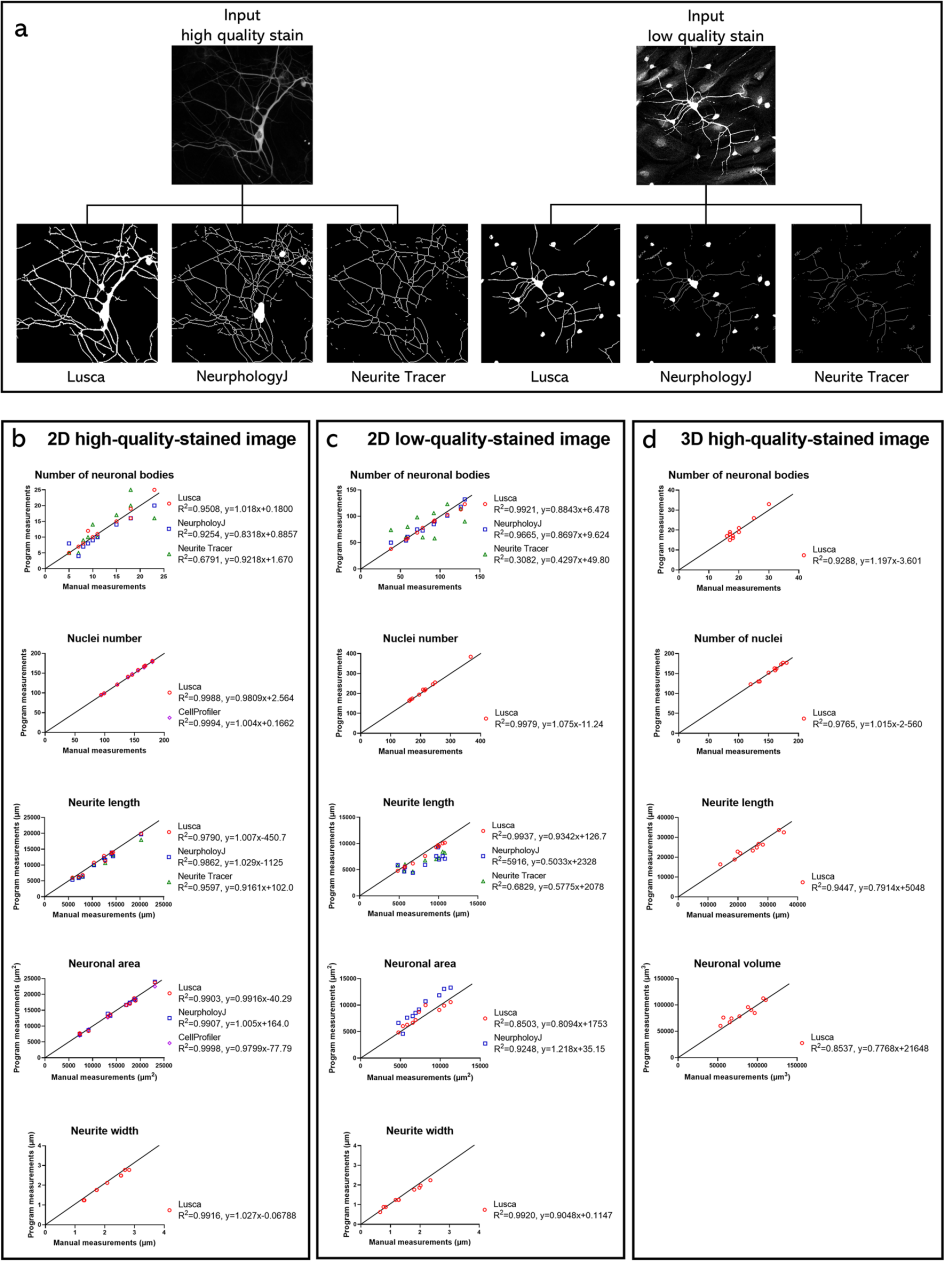
Qualitative and quantitative comparison of different scripts for neuron analysis with Lusca and manual tracing on 2D high and low-quality-stained and 3D images. Comparison between open-source programs, Lusca, and manual tracing performed on MAP2 high and low-quality-stained input images. (**a**) Qualitative comparison of Lusca, NeurphologyJ, and Neurite Tracer final output images for each image stain quality. Datasets (neuronal bodies and nuclear count, neurite length and width, and neuron area/volume) generated by each open-source program, Lusca and manual measurements subjected to linear regression for 2D (**b**) high and (**c**) low-quality-stained images, as well as (**d**) 3D images. The equations for the lines of best fit and the coefficients of determination are presented in the figure key.

Various tools facilitate analysis of neuronal morphologies, prompting a comparison of the results obtained with NeuronJ, NeuriteTracer, NeurphologyJ, and CellProfiler to those generated by both Lusca and manual measurements, the golden standard. Images of neurons were first analysed with ImageJ/FIJI scripts and compared accordingly (Fig. 2a). No notable difference was observed in length, soma and nuclei count, or neuron and nuclei surface area in both high and low-quality-stained images.

Further comparison included the calculation of execution speed (Table 1). For ten analysed images, NeuronJ measured 5 parameters in 222.76 min, Neurite Tracer assessed 2 parameters in 1.63 min, NeurphologyJ evaluated 15 parameters in 9.12 min and lastly, Lusca calculated 29 parameters in 6 min.

**Table 1:** Comparison of the available parameters within the three most used plugins for 2D neuron image analysis in ImageJ/FIJI to Lusca.

	ImageJ/Fiji Plugins			Lusca
	NeuronJ	Neurite Tracer	NeurphologyJ	
Nuclei number	✕	✕	✕	✓
Nuclei size	✕	✕	✕	✓
Nuclei shape	✕	✕	✕	✓
Nuclei intensity	✕	✕	✕	✓
Soma number	✕	✓	✓	✓
Soma size	✕	✕	✓	✓
Soma shape	✕	✕	✓	✓
Soma intensity	✕	✕	✕	✓
Neurite number	✕	✕	✓	✓
Neurite size	✕	✕	✓	✓
Neurite length	✓	✓	✓	✓
Neurite branching	✓	✕	✕	✓
Neurite width	✕	✕	✕	✓
Neurite intensity	✕	✕	✕	✓
Attachment points	✓	✕	✓	✓
Ending points	✓	✕	✓	✓
Colocalization	✕	✕	✕	✓
3D image analysis	✕	✕	✕	✓
Avg. execution speed per image (number of measurements/time*(min)*)	0.22	12.27	16.45	48.33

Additionally, linear regression was performed to compare the results of the aforementioned programs with manual measurements (Fig. 2b–d). Lusca was the only program to consistently mirror manual measurements throughout both 2D high and low-quality-stained images and 3D images.

**Figure 3:**
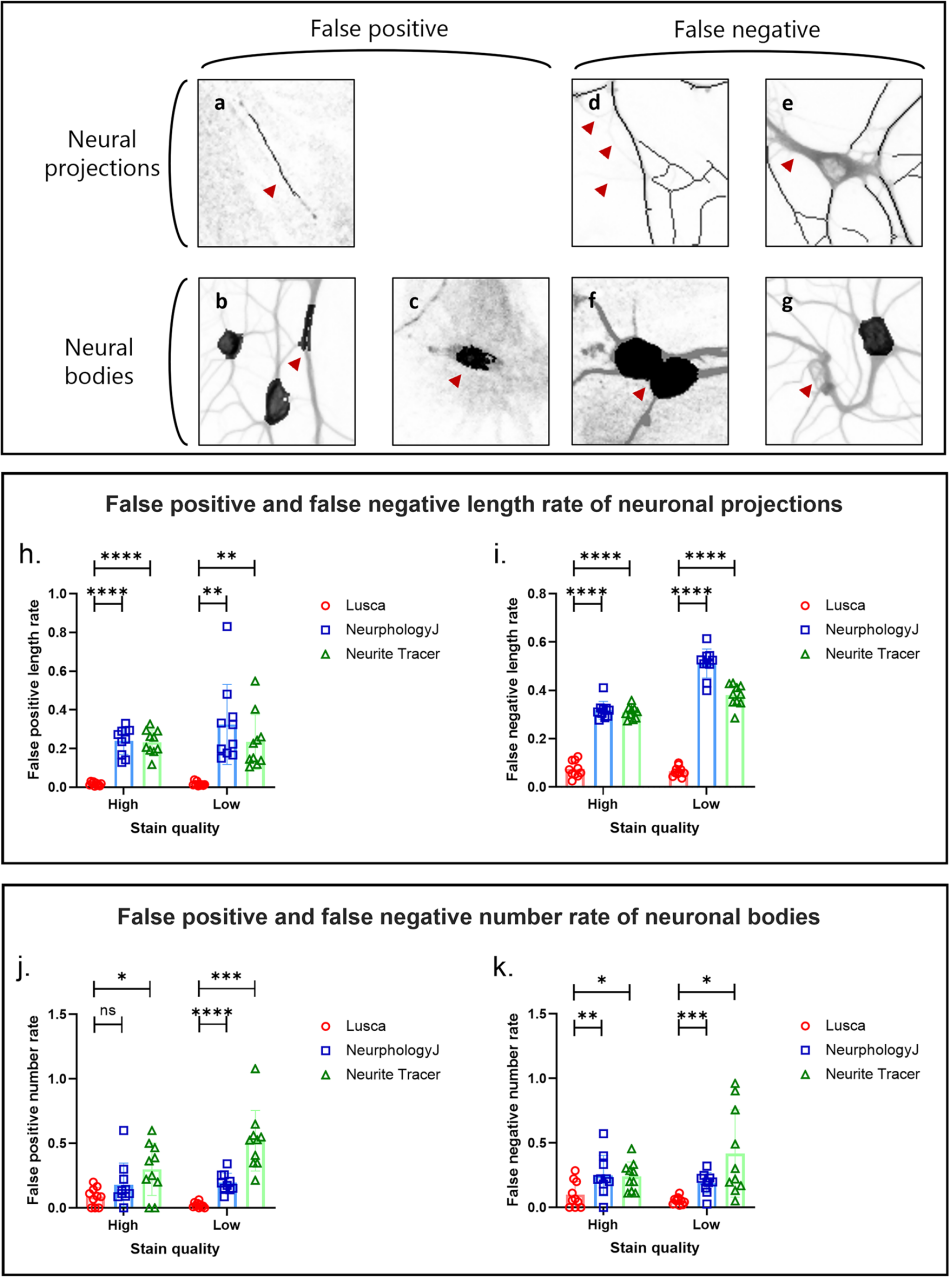
False positive and negative operational classifications and measurements rate comparison between NeurphologyJ, NeuriteTracer and Lusca. False negative signal defined as an unrecognized visible signal by the macro. False positive signal characterised by the macro but lacking an actual signal. Recognised false positive signals for neural projections: (**a**) background noise mistaken for neurite, while for neural bodies these include (**b**) thick neurite and (**c**) background noise misplaced for neural body. False negative signals for neural projections: (**d**) neurites recognised as background and (**e**) thick neurite misplaced to neural bodies, and for neural bodies (**f**) two connected neural bodies and (**g**) neural body recognised as background. For further comparison of ImageJ/FIJI macros, false positive and negative signals measured for each script on high and low-quality-stained MAP2 neuron images: false positive neurite length rate (**h**), false negative neurite length rate (**i**), false positive count rate of neuronal bodies (**j**), false negative count rate of neuronal bodies (**k**).

For each ImageJ macro (Lusca, NeuriteTracer, and NeurphologyJ) false positive and false negative measurements were calculated as well. Following Encarnacion-Rivera et al.’s study, false negative length was characterised by unrecognized visible neurite signals by the macro, while false positives were identified by the macro but lacked actual neurite signals^19^. False negative neuronal body count was defined as uncounted bodies with present signals, while false positives represented macro-recognized bodies lacking signal. False positive and negative rates were defined as measurements of neurite length or neuronal bodies divided by the manual measurements. Recognised false positive signals included background noise noted as a soma or a neurite signal and thicker neurite segment recognised as a neural body. On the other hand, false negative signals included merged neural bodies not recognised separately, neurite or neural body recognised as background due to low contrast, and neurite misplaced as neural body (Fig. 3a–g). Figure 3 shows that Lusca had significantly fewer false positive and negative length measurements compared to NeuriteTracer and NeurphologyJ in both high and low-quality-stained images. Similarly, Lusca showed fewer false positive and negative counts for neuronal bodies, a significant difference when compared to NeuriteTracer and NeurphologyJ. However, no difference was found for the false positive count in high-quality-stained NeurphologyJ images (Fig. 3j–k).

### Lusca and other cellular and subcellular structures

Besides neural morphometric analysis, Lusca successfully quantified various morphological parameters of blood vessels or mitochondria images.

### Application of Lusca in morphological analysis of blood vessels

**Figure 4:**
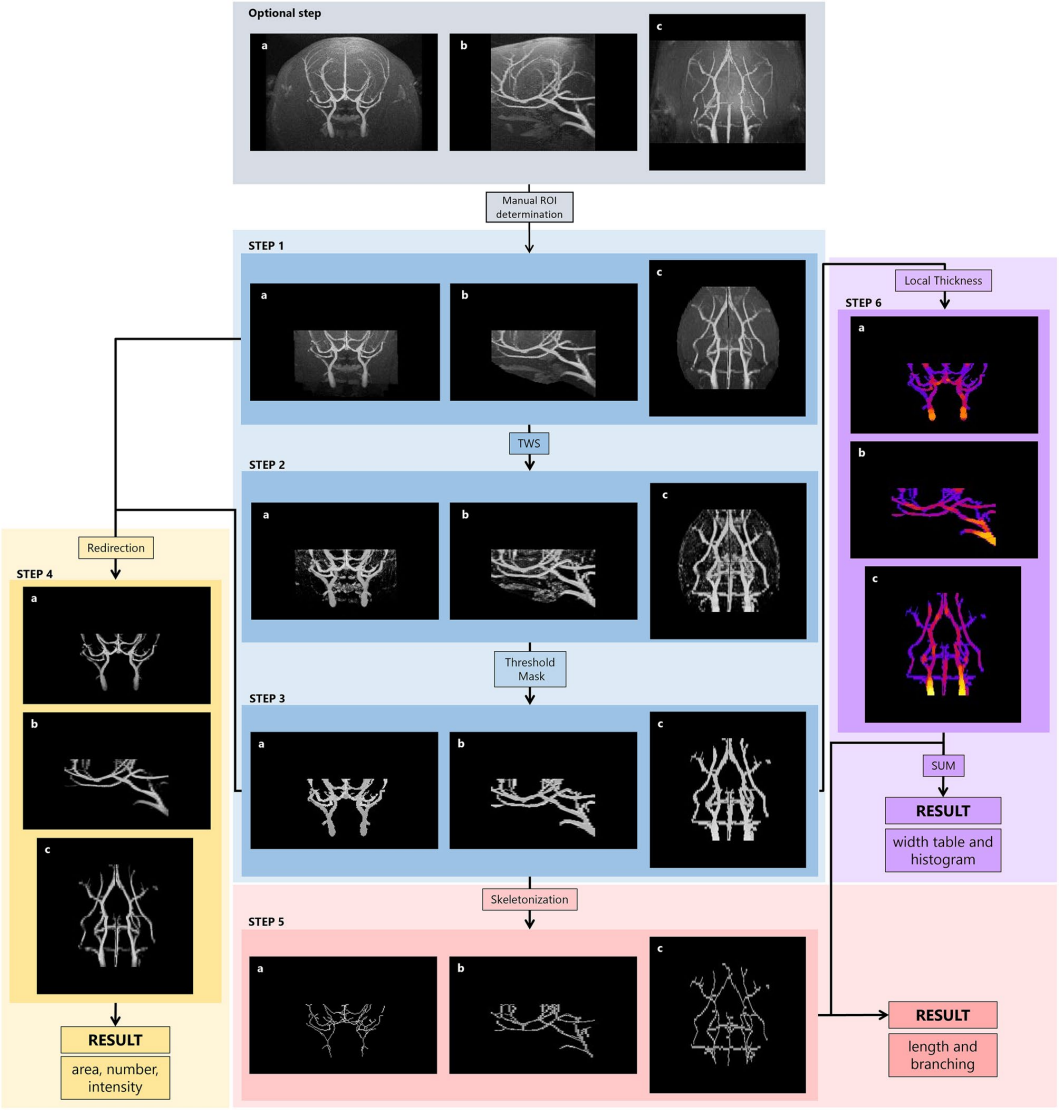
Lusca pipeline applied for morphological 3D magnetic resonance angiography (MRA) stack analysis. For 3D perception the anatomical planes (**a**) coronal, (**b**) sagittal and (**c**) transversal are shown. Blue, yellow, red, and violet squares represent image segmentation, area, number and intensity, length and branching, and width measurements respectively. The anatomical landmarks of MRA stacks used to standardize the volume and position of the maximum intensity projection ROIs (Optional step, grey square). Vessels on the input images in step 1 segmented from the background with TWS in step 2 and, after area and intensity thresholding, the step 3 segmented image is acquired. In step 4, after redirection of segmented image to the input image, the area, number, and intensity are obtained. For length and branching measurements, in step 3 the image undergoes skeletonization and analysis. Width is calculated by applying Local thickness mask on the image from step 3 to get step 6 image. Simultaneously, the threshold is applied to the 32-bit image from step 5, and after subtracting 255 values NaN for the background and 0 for skeleton pixels are obtained. Images from steps 5 and 6 are added to get step 7 image that serves for width calculations.

Since both neural projection and blood vessels are tubular objects, Lusca’s applicability in analysing 3D magnetic resonance angiography (MRA) image stacks was also tested^20^. Before analysis, anatomical landmarks were established to standardize the volume and position of maximum intensity projection ROIs (Fig. 4 Optional step). Though optional, this step ensures a more controlled analysis, since analysing the entire stack without such standardization might increase false positive areas due to MR coil-induced intensity variance. Volumetric vessel analysis was conducted using a specialized 3D classifier for distinguishing vessels from brain tissue and the background. Following the application of intensity and volume thresholds, results were obtained as measurements of vessel volume in the measured hemisphere (Fig. 4 steps 1–4). This confirmed Lusca’s ability to quantitatively assess high blood flow velocity cerebral vasculature in a longitudinal manner using MRA.

### Application of Lusca in morphological analysis of mitochondrial shapes

**Figure 5:**
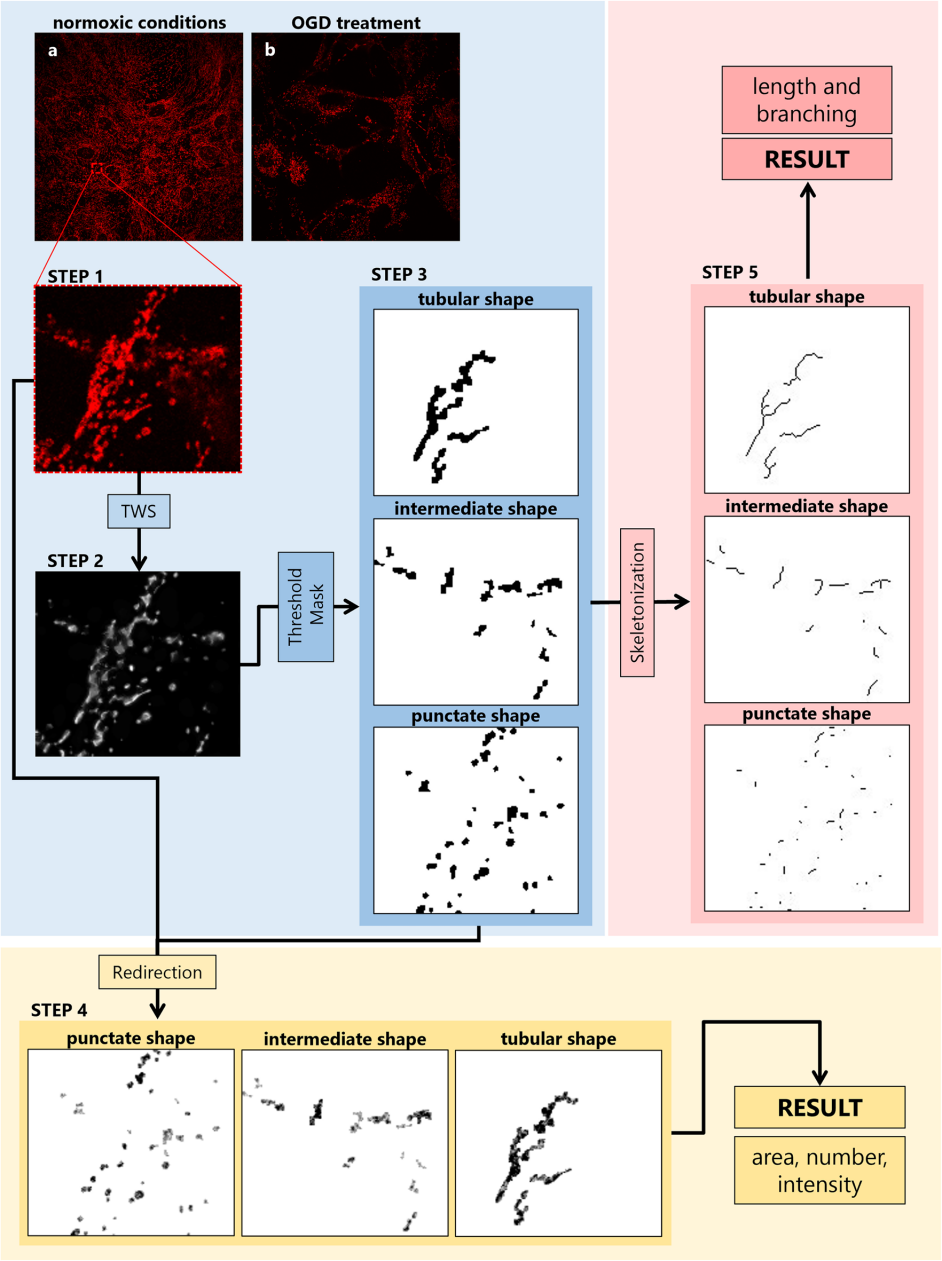
Lusca pipeline for morphological analysis of mitochondria. Blue, red, and yellow squares represent image segmentation, length and branching, and area, count and intensity measurements respectively. Mitochondria immunocytochemistry images in (**a**) normoxic and (**b**) after oxygen–glucose deprivation treatment were stained with Tomm20 antibody. Mitochondria (step 1) are segmented from the background with TWS in step 2 and after area, intensity and circularity thresholding, the step 3 image is acquired. In step 4 after redirecting the input image the result area, count and intensity are obtained. For further length and branching measurements, the step 3 image is skeletonized and analysed (step 5).

Subcellular structures like mitochondria compose distinct cellular networks. Morphological analysis of these networks provides insights into mitochondrial dynamics, quality, and function. Jagečić et al. demonstrated Lusca’s efficacy in comparing mitochondrial networks under varying cell conditions (Fig. 5a, b)^21^. A classifier was made to differentiate the mitochondrial network from the background (Fig. 5, steps 1–2). According to Ahmad et al., who described tubular, intermediate, and punctate mitochondrial shapes that were linked to functional changes, Lusca successfully segmented these shapes based on their configuration’s differences in circularity, categories of which included 0.00–0.33, 0.33–0.66, and 0.66–1.00 (Fig. 5, step 3)^22^. The analysis also included the calculation of area, number, intensity, length, and branching of the mitochondria (Fig. 5 steps 4–5).

## Discussion

Image analysis extracts information from images and, as such, represents an important part of biomedical research. Although there are open-source platforms available such as ImageJ/FIJI and CellProfiler, proprietary tools, like MATLAB and Imaris provide user-friendly interfaces alongside advanced and comprehensive tools. However, proprietary tools hide processing details, thereby limiting user interaction, which may not be desirable in biological research^23^. Therefore, an increase in the application of open-source platforms due to their transparency and accessibility can be noted.

Even though manual neuron analysis is the golden standard in image analysis, it is time-consuming and usually represents a substantial bottleneck for elaborate projects. Moreover, such a method carries inherent bias. Even if we exclude the differences in manual image analysis among different scientists, there is still a possibility that each processed image would not be analysed utilizing the same criteria. As computer technology advances daily, there's a push towards automatization of image analysis.

Machine learning, a form of artificial intelligence, predicts outcomes from gathered experience. So far it has been applied in various scenarios in biomedical sciences, including the development of therapeutic targets, drug discovery as well as the making clinical and/or pathological diagnoses from medical and/or histological images^24–28^. Image analysis using machine learning segmentation of biological structures relies on patterns set by the user and is capable of distinguishing between pixels that share certain similarities, going beyond simple intensity analysis through thresholding toward detailed analysis of morphological features. To harness this technological development, ImageJ/FIJI includes TWS, a machine learning-based plugin with a user-friendly interface and improved image segmentation capabilities^13^. Although thresholding paired with filtering provides faster segmentation in tools like Neurite Tracer and NeurphologyJ, it leads to imprecise results in regions of similar intensity as well as low-intensity and noise-rich images. Hence, by integrating TWS in Lusca, augmentation of precision, enhancement of noise/artifact elimination, detail recognition, accurate object delineation, and wider application possibilities are achieved (Figs. 2, 3, 4, 5). The advantage of Lusca as an optimal solution for image segmentation is best demonstrated in Fig. 3. Therein it is visible that Lusca outperforms both NeurphologyJ and Neurite Tracer in terms of accuracy. This is evidenced by the statistically lower rates of false positives and negatives in both the length and number of neuronal bodies on both high- and low-quality-stained images. However, it is important to note that there was no statistical difference observed between Lusca and NeurphologyJ in terms of the false positive rate for the number of neuronal bodies on high-quality-stained images.

Another advantage of Lusca is its fully automated interface, making it time efficient. Moreover, since minimal user input is needed for setting of the parameters before image analysis, Lusca provides a more objective, and standardized procedure that can easily be replicated by other researchers. Generally, the speed of image analysis depends on image size, chosen features for training classifiers, and the number of cores on the computer running FIJI. NeuronJ, a semi-manual plugin, accelerates neural projection tracing as compared to manual analysis, however, it is still time-consuming and retains inherent biases^5^. Therefore, NeuriteTracer and NeurphologyJ, even faster, less biased and automated software were created^10,11^. NeuriteTracer still requires manual addition of background, images of both nuclei and neurite markers, and the wizard is separated from the image analysis part of the programme, so it requires restarting the program. On the other hand, NeurphologyJ requires extensive user input both at the initiation of the program, including entering input values within the wizard, and at the end to obtain the results. Furthermore, NeurphologyJ also demonstrated issues during the setup and initiation of the high throughput version. Because of this, our comparison utilized NeurphologyJ’s interactive version, requiring restarting the program prior to each image analysis. Ultimately, since both of these programs demonstrate a frequent need for user input, including rebooting and re-entering parameters for each image, attempts at automated analysis become demanding and time-consuming.

Lusca also provides a comprehensive array of quantifiable variables, surpassing other programs for morphological analyses (Table 1). Since there is a proportional relationship between the number of obtainable measurements and the duration of analysis, it was insufficient to directly compare the program’s analysis time as different algorithms measure various parameters. Consequently, the average execution speed was defined and calculated for each algorithm. Despite longer single-image analysis compared to Neurite Tracer and NeurphologyJ, Lusca’s broader measurement options enable extensive automated analysis, resulting in the highest execution speed. Additionally, Lusca’s capability for 3D image analysis distinguishes it from other software (Table 1, Figs. 2 and 4). Beyond wider quantification options, Lusca also allows users to analyse specific image sections and, unlike NeurphologyJ and CellProfiler, set the desired scale, facilitating targeted analysis.

Lusca offers a user-friendly wizard, guiding new users through the image analysis process by displaying a preview of the particular pipeline step based on input intensity, area/volume and circularity thresholds. Its interactive threshold adjustment achieved using “while” loops, also allows fine-tuning for satisfactory segmentation. Although parameter setting might take longer when compared to other software, Lusca’s extensive measurements compensate for the initial time spent, ensuring a comprehensive output.

Additionally, even though an attempt was made to compare Lusca’s performance to AutoNeuriteJ, a more recent image analysis programme, issues were encountered regarding the neuron analysis that extend up to the image edge because of the default “exclude on the edge option” setting^12^. Although suitable for measurements of individual neuron morphology, this setting limits AutoNeuriteJ and renders it unsuitable for certain image types. This includes images capturing high-magnification neuronal cultures, older neuronal cultures where neuron size drastically increases compared to younger cultures, neuronal cultures with visible neural synapses or overlapping neural projections as well as histological slides of brain tissue. To facilitate both types of analyses, Lusca offers an adjustable area/volume threshold and an edge exclusion option.

Lusca’s limitations involve the creation of new classifiers for different objects or varying object features, such as younger and older neuron cultures and IHC images with altered illumination. Lusca’s analysis speed is mostly influenced by image quality, selected training features in TWS, and CPU power. For future experiments, the utilisation of GPU acceleration to expedite the analysis of complex images is planned.

By applying machine learning in Lusca’s pipeline, classifiers for various biological objects could easily be generated. So even though Lusca was developed for easy, fast, and accurate analysis of neural projections and bodies as described here, additional extensive studies proved that our algorithm could also be extended for use in the quantification of both mitochondrial and vessel morphology (Figs. 4 and 5)^20,21^. Consequently, recognizing its broad applicability, quantification modules facilitating separate measurements of length and branching, width, count, area/volume, and intensity were added to neuronal projections and bodies quantification (Fig. 1. red, violet, and yellow square). So far, we have effectively applied this method to analyse 3D MRA vessel images and Tomm20 immunofluorescent images for mitochondria.

We wanted to examine whether Lusca could be applied for 3D MRA vessel analysis since it would have allowed us to clarify the differences in the cerebral vascular perfusion status between the Koizumi and Longa middle cerebral artery occlusion, preclinical stroke models, monitored by longitudinal in vivo MR imaging^29,30^. Due to the resolution of the MR scanner and the chosen sequence, we were unable to analyse the changes in the capillary network or discern between complete vessel obstruction and hypoperfusion. However, Lusca enabled us to reliably measure the ischemia-induced changes in arterial vasculature volume reflecting the vascular perfusion status of the mouse brain. This long-term analysis allowed us to redefine the Koizumi method as a chronic hypoperfusion ischemia model and the Longa method as an ischemia and reperfusion model^20^.

Additionally, Lusca was also tested for morphological analysis of mitochondria following oxygen–glucose deprivation (OGD), the most used model to study ischemic stroke in vitro. The morphology of mitochondria was divided into tubular, intermediate and fragmented shapes, depending on fusion and fission cycles in cells^22^. The analysis helped us reveal the change in the total Tomm20 positive area in maturing neuronal stem cells compared to the control group, and assess the different ratios of mitochondrial shapes in both groups^21^.

Based on the aforementioned, promising experiments with morphological analysis of mitochondria and vessels, we believe that Lusca could perform many other image analysis tasks pertaining to biological objects, including capillary or lymph vessel area/volume quantification, length and width, the area/volume of the cellular cytoskeleton or cellular organelles measurement, and obtaining of the number of cells which are immunohistochemically positive for malignant biomarkers. All of this further fortifies the idea that Lusca could become a promising script for image analysis for researchers in biomedical fields using FIJI (ImageJ).

## Conclusion

Lusca, a new FIJI (ImageJ) tool, enhances image segmentation and analysis using a machine learning algorithm and existing FIJI (ImageJ) plugins. Its user-friendly interface and precise measurements offer an accessible alternative to both open-source and proprietary programmes. Fully automated and high throughput, Lusca accurately quantifies diverse parameters and can analyse various biological structures. Its automated analysis saves time and facilitates the standardisation of protocols for easy sharing among researchers, enhancing analysis reproducibility and research facilitation.

## Methods

### Neural cell culture

Neural stem cells were isolated from the telencephalic wall of 14-days-old mice embryos, and then grown in suspension in a proliferation medium composed of DMEM/F12, 1% Pen/Strep, 0.5% Hepes, supplemented with 1% N2, 2% B27 and growth factors (comprising 0.2% EGF, 0.2% FGF-basic). As these cells multiplied, they formed neurospheres, that were dissociated when reached 150–200 µm in diameter. For this experiment, we used cells in passage 3 (P3) which were seeded for differentiation on poly-D-lysine and laminin coated wells in a medium for differentiation. The differentiation medium comprised the same elements as the proliferation one, excluding the growth factors, and including 1% heat inactivated FBS, 2% of B27 plus instead of B27. Following 4 days of growth in the differentiation medium, DMEM/F12 was replaced with Neurobasal, and was changed every 3 days. On days 5, 7, and 10 of differentiation, the cells were fixed, ICC method was performed and expression of markers of mature neurons (MAP2, β3-tubulin) and astrocytes (GFAP) were analysed.

### Immunocytochemistry

For immunofluorescent staining, the cells were firstly permeabilized with 0.2% Triton X-100 in PBS for 10 min after which they were blocked in 3% donkey serum in PBS for 1 h at room temperature (RT). Primary antibodies (anti-MAP2 1:1000, Abcam; and anti-SMI312 1:200, Biolegend) were diluted in 3% donkey serum in PBS and incubated overnight at 4 °C. Following washes with PBS, secondary antibodies (Alexa Fluor 488 1:1000; and Alexa Fluor 546 1:1000, Life Technologies) were diluted in 1% donkey serum in PBS and incubated for 2 h at RT. Cells were gently washed with PBS and counterstained with DAPI for 10 min at RT, washed again and mounted with DAKO fluorescent mounting medium. As negative controls for all antibodies, secondary antibody-only controls were carried out.

### Prerequisites for image analysis with Lusca

The Lusca script, as well as the User manual, example images for each category (2D Channel images, 2D Single images, 3D Channel images, 3D Single images), classifiers and document with input data for test image analysis, can be freely downloaded from GitHub (https://github.com/MediSciScripter/Lusca). Once Lusca is installed, it’s important to verify whether the version of FIJI (ImageJ) includes the LocalThickness; if not, proceed with the installation of the plugin prior to running Lusca. Furthermore, the user should also include additional update sites such as Neuroanatomy and ImageScience (Help → Update → Manage update sites) into their programme. Installing Neuroanatomy enables the user to summarise the results from skeleton images, while ImageScience provides the user with features like Derivates, Laplacian and Structure for TWS 3D. All images and classifiers for the analysis should be placed into their corresponding folders (one folder of images and one for classifiers).

### Validation of Lusca with manual measurements

The manual counting of the number of neural bodies and nuclei was performed using the CellCounter. Each soma and nuclei on high and low-quality-stained 2D images and 3D images were manually marked while the CellCounter was counting the number of markers.

A macro with thresholding and object area measurement was developed to simplify time-consuming manual area measurements since manual measurement would be impossible for evaluating delicate neural projections. Initially, a semi-automated macro with a threshold of 20–255 was set and tested on 10 high-quality nuclei images. There was no statistical difference in the semi-automated macro and manual measurements using the freehand tool in ImageJ/FIJI. This macro was then used to measure the area for both high and low-quality neural images, with adjusted thresholds (20–255 for high-quality and 200–255 for low-quality) to minimize background noise while capturing accurate neuron area measurements. Similarly, for volumetric manual measurements, a simple macro with thresholding (intensity of 11–255 for 8-bit image and volume 350-Infinity pixels) and stack statistic volume measurements was utilized.

The manual measurement of length for 2D images was performed using the freehand line tool in ImageJ/FIJI. All the traces were saved to the ROI manager and later compared to the traces obtained with Lusca. For 3D images, length was manually measured by skeletonising and analysing threshold objects obtained from the volumetric analysis.

Width measurements for manual validation were performed with the straight line. All 20 width ROIs (one for each 2D high and low-quality-stained image) were saved to the manager. Traces were then compared to Lusca measurements using the images like Fig. 1 step 7 since the width result macro made needed to be on the same location as manual measurements. When locating the pixel whose value corresponded to the width macro measured, its value was noted.

### Image analysis parameters for comparison of Lusca to similar software

NeuronJ is a FIJI (ImageJ) plugin that offers manual tracing of neural projections while automatically updating the cursor to follow the estimated path determined by an increased intensity of the neural projections^5^. The measurements were performed as described on both high and low-quality-stained images.

Neurite Tracer is a mainly automated ImageJ plugin that consists of two separate parts that are run by the user^10^. The first part is semi-manual and consists of a wizard that enables the user to select parameters for the analysis. The second part consists of setting the input parameters and a fully automated image analysis. The parameters for the analysis of high-quality-stained images were set as follows: lower threshold on neuronal images 50, lower threshold on nuclear images 40, image-scale (pixel/micron, 1 for pixels) 1.6090599, size of smallest nuclei 120, size of largest nuclei infinity. Input parameters for low-quality-stained images were lower threshold on neuronal images 120, lower threshold on nuclear images 30, image-scale (pixel/micron, 1 for pixels) 1.6090599, size of smallest nuclei 100, size of largest nuclei infinity. For both image types, prefix for neuronal images “Neuron”, and prefix for nuclear images “Nuclei”.

NeurphologyJ is a fully automated algorithm for neural analysis in FIJI (ImageJ)^11^. It requires minimal user input during initialisation and end of the image analysis. For high-quality-stained images, the input included: contrast level threshold 7, soma intensity threshold 20, width of neurite in pixels 5, particle clean-up value 3. The threshold setting towards the end of the image analysis was set to 7 for neural projections and 7 for the images of the soma. For low-quality-stained images, the image parameters were set to 60 for contrast lever threshold, 120 for soma intensity threshold, 5 for width of neurite in pixels, and 5 for particle clean-up value. The threshold for soma images was set to 170, while for neural projections was set to 180.

Since the module “MeasureNeurons” was no longer available with our 4.0.0. version of CellProfiler, for the image analysis a simple pipeline was created for measurements of neuron area, nuclei area, and nuclear number^31,32^. Input parameters for high-quality-stained images were adjusted manually to resemble the manual analysis in ImageJ/FIJI since similar parameters were needed for both analyses. Namely, the threshold was set to 0.078, which corresponds to manual measurements of 20–255 (20 divided by 255), and the area minimum and maximum value were set to 6 with 500 pixels for nuclei and 6 and infinity pixels for the surface of a neuron. After manually assembling the pipeline and setting of measurements, the images were analysed automatically.

### Volumetric vessel analysis

Gradient-echo images were acquired using a 3D-FLASH sequence on a 7 T MR system. Anatomical landmarks were used to standardize the volume and position of the maximum intensity projection ROIs. Volumetric vessel analysis was performed on the final images of the objects with Lusca and expressed as vessel volume of the measured hemisphere. A detailed breakdown of the methodology can be seen in the paper by Justić et al.^20^.

### Morphometric analysis of mitochondria

Neural stem cells isolated from the telencephalic wall of 14.5 days old mouse embryos were cultivated in differentiation medium. On days 1, 7 and 14 of differentiation cells were exposed to 24 h long oxygen glucose deprivation treatment. Immunocytochemistry was performed using Tomm20, a mitochondrial outer membrane marker. Lusca was used to analyse mitochondrial morphology. A detailed breakdown of the methodology can be seen in the paper by Jagečić et al.^21^.

### Statistical analysis

The raw data were compiled and categorized into corresponding groups. The width parameter, number of nuclei for 2D low-quality-stained images, and all parameters for 3D images (volume, length, number of neuronal bodies, and nuclei) were analysed using a paired t-test. All other statistical analysis was done using a one-way ANOVA with a Tukey Kramer post-hoc test to characterize specific differences between the groups. The statistical analysis of false positive and false negative values was performed using two-way ANOVA with Greenhouse–Geisser (GG) correction and Tukey post hoc test. The significance levels are as follows: * *p* < 0.05, ** *p* < 0.01, *** *p* < 0.001, **** *p* < 0.0001. Statistical analysis was conducted using GraphPad Prism version 9.3.1 (GraphPad Software, La Jolla, CA, USA). All the results are represented as a mean and SD.

### Ethical approval and consent to participate

All experiments on animals described in this work received approval of the Internal Review Board of the Ethical Committee of the School of Medicine, University of Zagreb: 380-59-10,106-17-100/27 received on 26.01.2017. All methods were carried out in accordance with relevant guidelines and regulations (Directive 2010/63/EU). All methods are reported in accordance with ARRIVE guidelines (https://arriveguidelines.org) for the reporting of animal experiments.

### Supplementary Information

Supplementary Information 1.

Supplementary Information 2.

## Data Availability

All data generated or analysed during this study are included in this published article and its supplementary information files. The script is available on GitHub (https://github.com/MediSciScripter/Lusca).
