# Author Correction: Lusca: FIJI (ImageJ) based tool for automated morphological analysis of cellular and subcellular structures

**DOI:** 10.1038/s41598-024-59795-w

**Published:** 2024-04-19

**Authors:** Iva Šimunić, Denis Jagečić, Jasmina Isaković, Marina Dobrivojević Radmilović, Dinko Mitrečić

**Affiliations:** 1 Department of Histology and Embryology, University of Zagreb School of Medicine, 10000 Zagreb, Croatia; 2 Laboratory for Stem Cells, Department for Regenerative Neuroscience, Croatian Institute for Brain Research, University of Zagreb School of Medicine, 10000 Zagreb, Croatia; 3 School of Medicine, European University Cyprus – Frankfurt Branch, 60488 Frankfurt am Main, Germany; 4 Laboratory for Regenerative Neuroscience, Department for Regenerative Neuroscience, Croatian Institute for Brain Research, University of Zagreb School of Medicine, 10000 Zagreb, Croatia

Correction to: *Scientific Reports* 10.1038/s41598-024-57650-6, published online 28 March 2024

The original version of this Article contained errors in the names of the authors Iva Šimunić, Denis Jagečić, Jasmina Isaković, Marina Dobrivojević Radmilović & Dinko Mitrečić, which were incorrectly given as Šimunić Iva, Jagečić Denis, Isaković Jasmina, Dobrivojević Radmilović Marina & Mitrečić Dinko.

The original Article has been corrected.

