# Supplementary material for: Lusca: FIJI (ImageJ) based tool for automated morphological analysis of cellular and subcellular structures
Source: Sci Rep. 2024 Mar 28;14:7383. doi: 10.1038/s41598-024-57650-6 (PMC10978859; doi:10.1038/s41598-024-57650-6)
Supplement: Supplementary file 1 — Supplementary Information 1. [file 41598_2024_57650_MOESM1_ESM.docx]

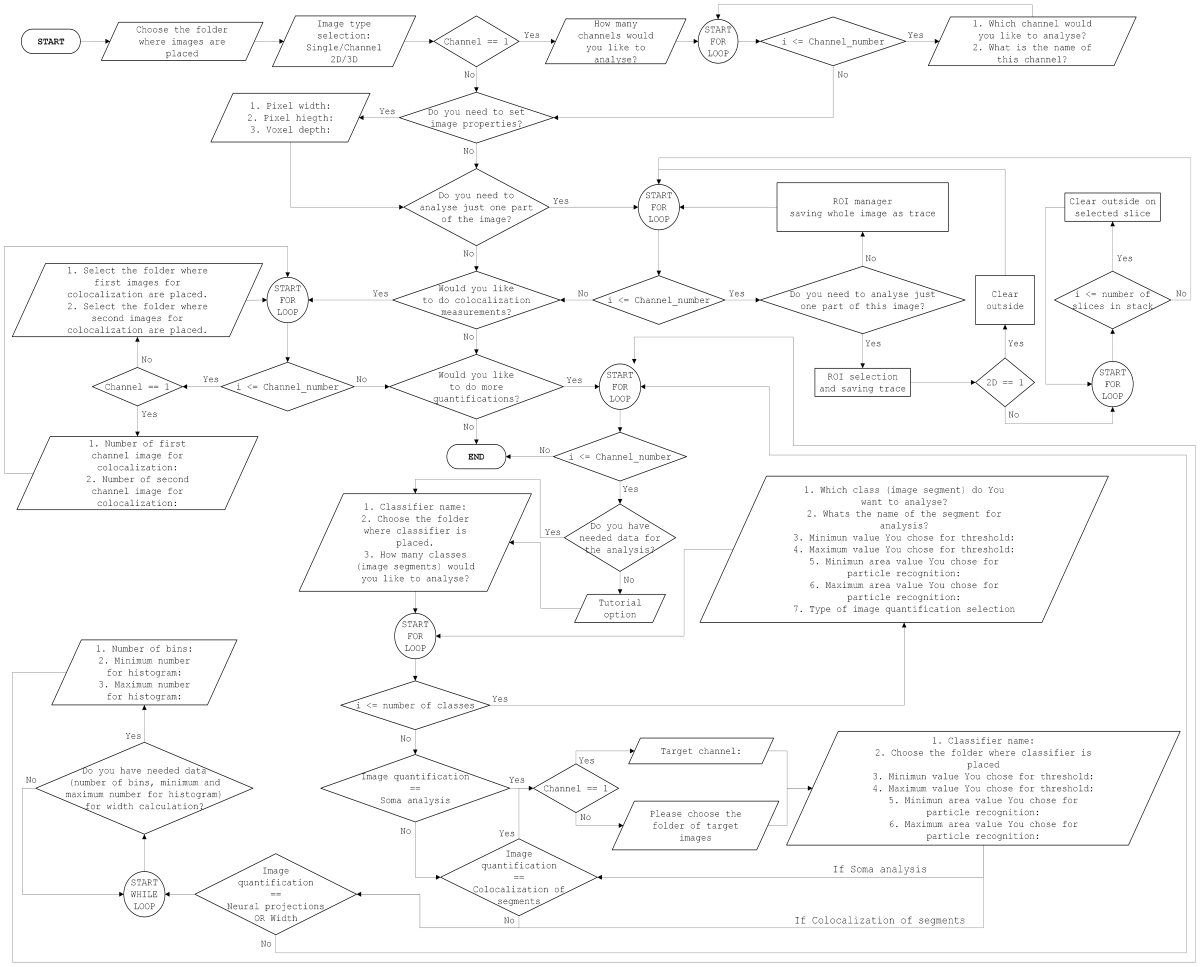


**Figure S1.** Flowchart of the settings which the user can select within the Lusca architecture.

|  | **Manual** | **NeuronJ** | **NeurphologyJ** | **NeuriteTracer** | **CellProfiler** | **Lusca** |
| --- | --- | --- | --- | --- | --- | --- |
| **Number of neuron bodies** | 12.40 ± 5.816 | **/** | 11.20 ± 5.029 | 13.10 ± 6.506 | **/** | 12.80 ± 6.070 |
| **Number of nuclei** | 144.9 ± 31.40 | **/** | **/** | **/** | 145.7 ± 31.54 | 144.7 ± 30.82 |
| **Nuclei area** | 134.6 ± 39.69 | **/** | **/** | **/** | 133.3 ± 40.28 | 133.6 ± 40.60 |
| **Neuron area** | 14636 ± 5409 | **/** | 14552 ± 5464 | **/** | 14264 ± 5301 | 14473 ± 5390 |
| **Neurite length** | 11925 ± 4258 | 11315 ± 4174 | 11145 ± 4412 | 11027 ± 3982 | **/** | 11558 ± 4334 |
| **Neurite width** | 2.003 ± 0.6094 | **/** | **/** | **/** | **/** | 1.990 ± 0.6288 |

**/** Parameter is not readily available in the programme.

**Table S1.** Mean and SD of analysed parameters for 2D high-quality-stained images of neurons measured with NeuronJ, NeurphologyJ, NeuriteTracer, CellProfiler and Lusca

|  | **Manual** | **NeuronJ** | **NeurphologyJ** | **NeuriteTracer** | **Lusca** |
| --- | --- | --- | --- | --- | --- |
| **Number of neuron bodies** | 85.40 ± 30.55 | **/** | 83.90 ± 27.02 | 86.50 ± 23.65 | 82.00 ± 27.12 |
| **Number of nuclei** | 222.3 ± 65.83 | **/** | **/** | **/** | 223.9 ± 64.39 |
| **Neuron area** | 7643 ± 2252 | **/** | 9346 ± 2853 | **/** | 7939 ± 1977 |
| **Neurite length** | 8227 ± 2288 | 7780  2202 | 6469 ± 1497 | 6829 ± 1599 | 7813 ± 2144 |
| **Neurite width** | 1.413 ± 0.5913 | **/** | **/** | **/** | 1.393 ± 0.5372 |

**/** Parameter is not readily available in the programme.

**Table S2.** Mean and SD of analysed parameters for 2D low-quality-stained images of neurons measured with NeuronJ, NeurphologyJ, NeuriteTracer and Lusca

|  | **Manual** | **Lusca** |
| --- | --- | --- |
| **Number of neuron bodies** | 19.80 ± 4.417 | 20.10 ± 5.486 |
| **Number of nuclei** | 154.7 ± 19.72 | 154.5 ± 20.26 |
| **Neuron volume** | 81453 ± 20460 | 84917 ± 17200 |
| **Neurite length** | 24924 ± 6666 | 24772 ± 5427 |

**Table S3. Mean and SD of analysed parameters for 3D images of neurons measured manually and with Lusca**
