# Supplementary material for: Lusca: FIJI (ImageJ) based tool for automated morphological analysis of cellular and subcellular structures
Source: Sci Rep. 2024 Mar 28;14:7383. doi: 10.1038/s41598-024-57650-6 (PMC10978859; doi:10.1038/s41598-024-57650-6)
Supplement: Supplementary file 2 — Supplementary Information 2. [file 41598_2024_57650_MOESM2_ESM.pdf]

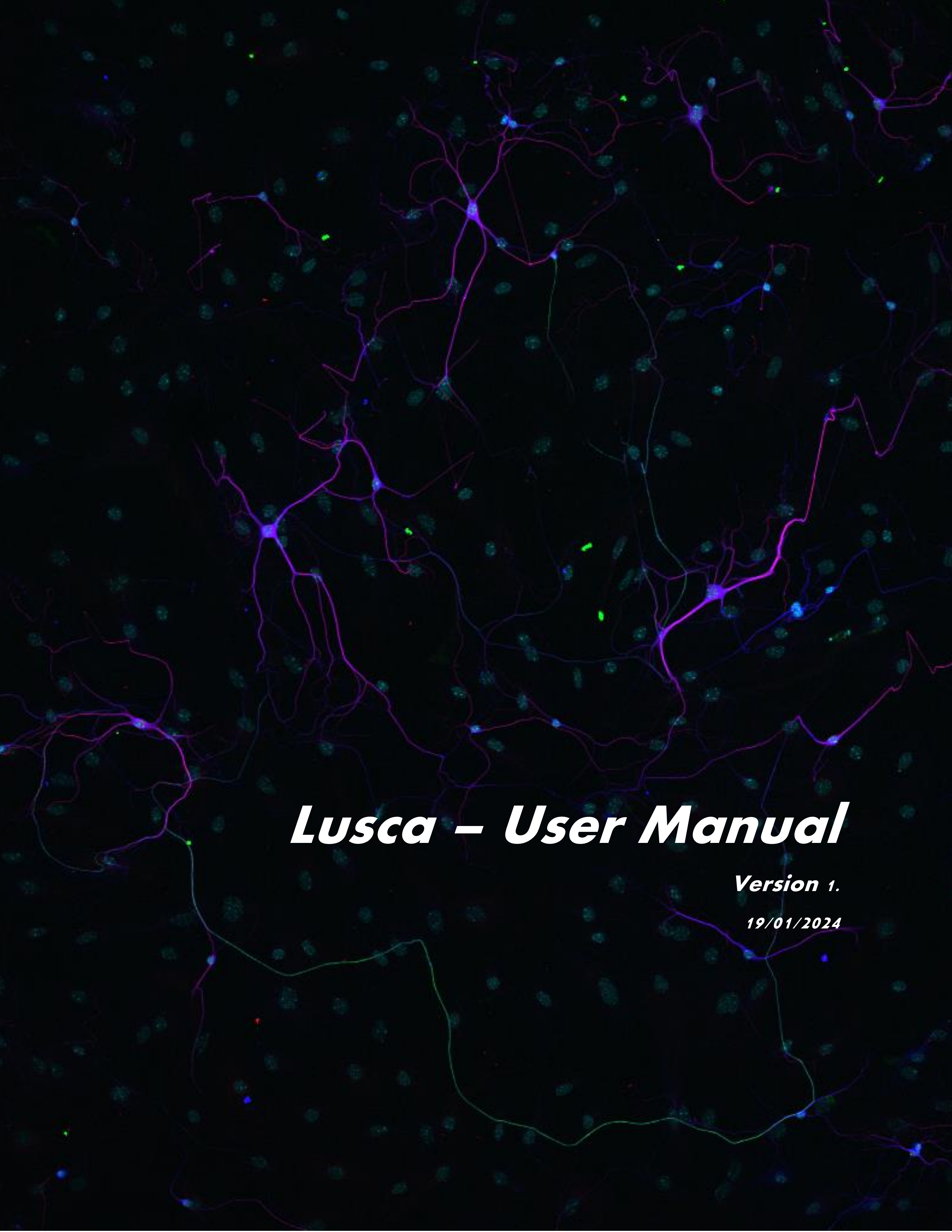

Lusca – User Manual

Version 1.

19/01/2024

Table of Contents

1. **Introduction** 1
2. **Getting started** 1
   2.1. **Set-up, installation and accessing the macro** 1
   2.2. **Macro organization** 3
   2.3. **The input parameters** 4
      2.3.1. **Image folder** 4
      2.3.2. **Image type, setting scale, cropping and morphological analysis** 4
      2.3.3. **Classifier(s) folder** 6
      2.3.4. **Analysis for the first batch of images – Interactive part of the macro** 6
      2.3.5. **Analysis for the other batches of images – only the input of the parameters** 12
      2.3.6. **Image analysis without image segmentation** 15
   2.4. **The morphological parameter results** 16
   2.5. **End of the analysis** 17
3. **Troubleshooting and support** 17
4. **Citation** 17
5. **License** 17

1. Introduction

This macro implements a machine learning tool for image segmentation, which enables better segmentation of pixels for the analysis of noisy images or images with low contrast of desired objects compared to the background. Furthermore, it offers many possibilities for morphological image analysis (area or volume, number, intensity, length, width, and colocalization of segmented objects). Due to the special procedure of image segmentation, it is also capable of analyzing other biological structures recorded with different techniques (immunocytochemistry, MRA, in vitro cultures), such as blood vessels, and cellular organelles.

2. Getting started

2.1. Set-up, installation and accessing the macro

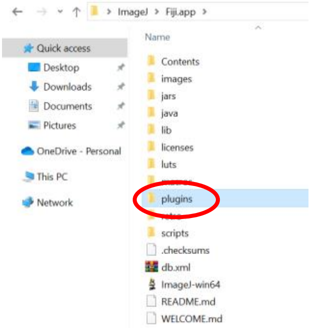

- Download the file "Lusca_PC.ijm" or "Lusca_MAC.ijm" from GitHub depending on the operating system of your computer.
- Put the macro file into the folder "plugins" of FIJI.

- Restart FIJI. Lusca should appear at the bottom of the Plugins menu.

- Before using Lusca, please check and install if necessary, the plugin "LocalThickness".

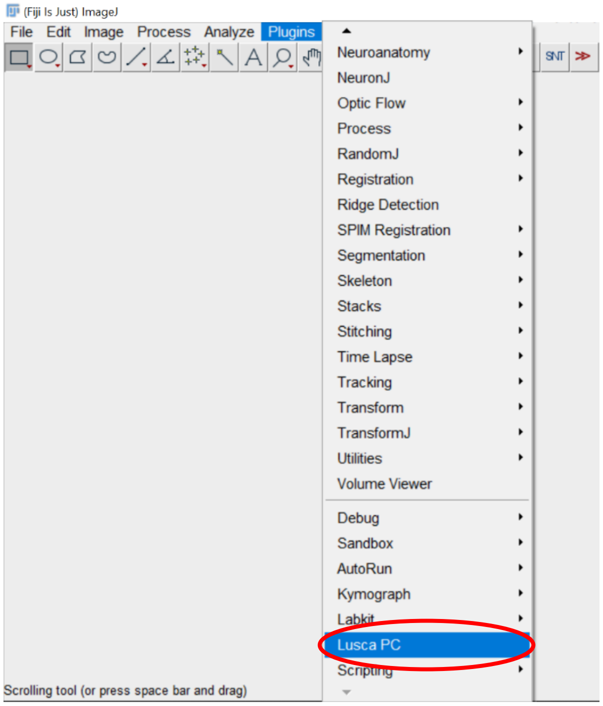

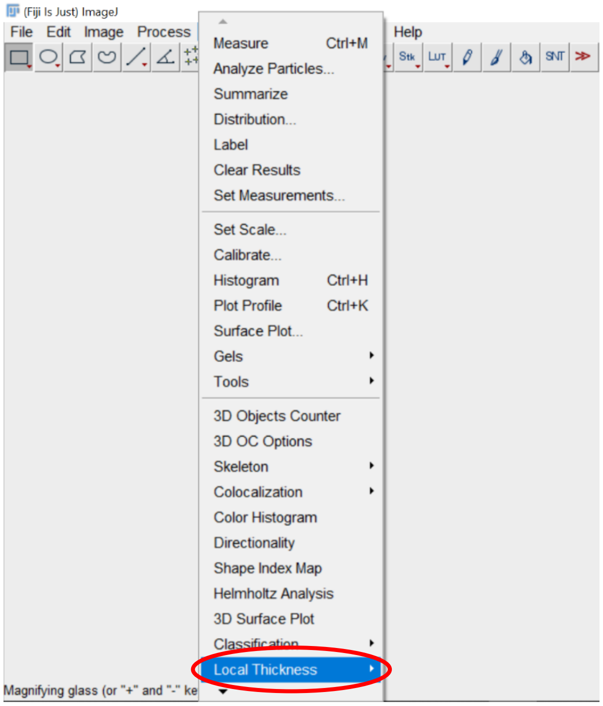

- Before using Lusca, please add the update sites “Neuroanatomy” and “ImageScience” in FIJI.

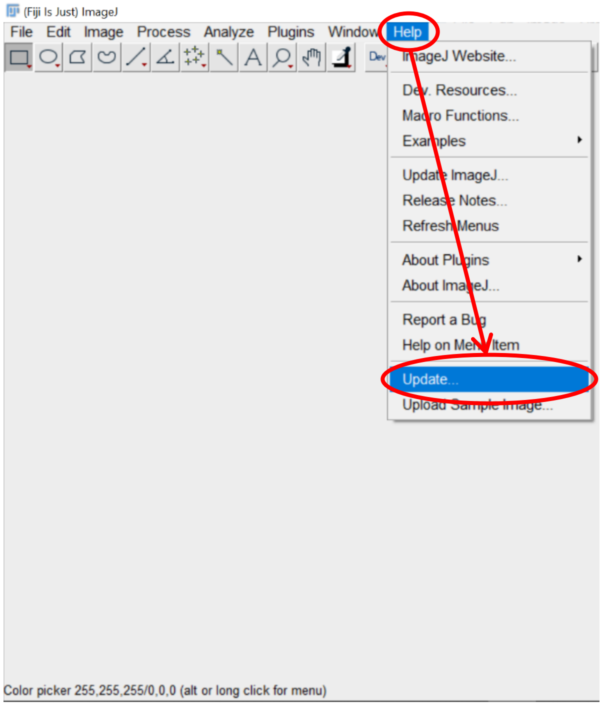

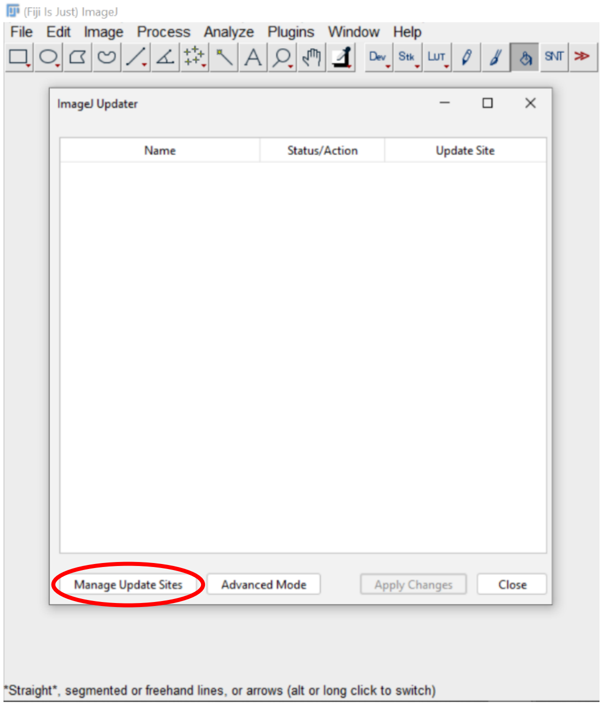

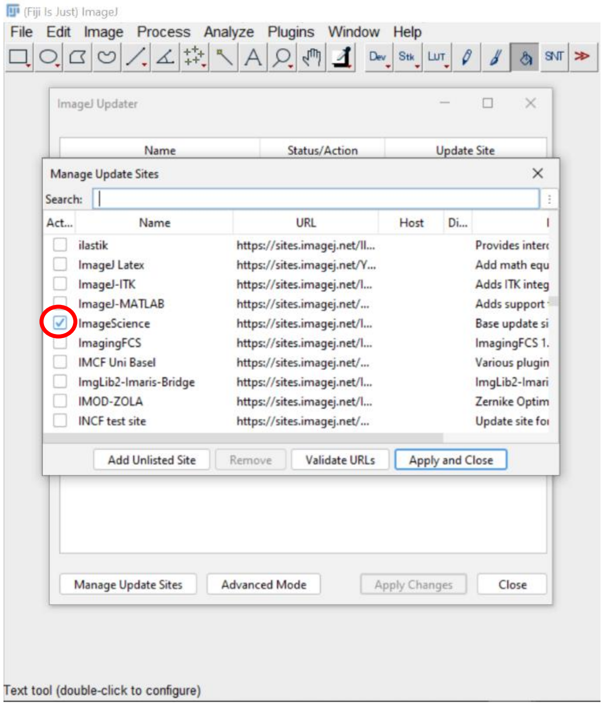

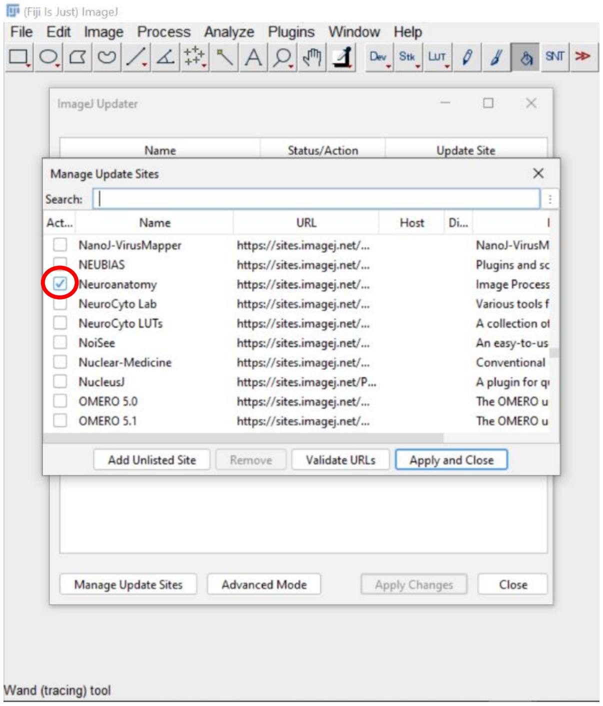

2.2. Macro organization

The image analysis by Lusca could be divided into two mayor parts considering the need for user:

1. Input of parameters – controlled by the user,
2. Image analysis – fully automated step controlled by selected input parameters.

Since the "Input of parameters" part of the macro is controlled by the user and by it the image analysis process depends, here only that part of the macro will be described.

The wizard guides users through the selection of input parameters which include:

1. **obligatory – the user has to enter these parameters,**
    a) image folder,
    b) image type (e.g., channel/single, 2D/3D), and optionally setting scale, cropping the image for analysis and/or the user can proceed to quantify other morphological parameters with or without interactive approach, or without image segmentation
    c) classifier(s) folder,
    d) the name of the image segmentation classifier and the total count of classes that will be analyzed,
    e) the number and name of each class that will be analyzed,
    f) the intensity, area/volume, and circularity thresholds for fine-tuning for each class,
    g) the type of morphological analysis (*neural projections, soma, area, number and intensity, length and branching, width, and colocalization of segments*) for each class,

2. **optional – the input of these parameters depends on the chosen morphological analysis,**
    h) histogram parameters (number of bins, minimum and maximum width) for *neural projections* or *width analysis*,
    i) parameters from d) to f) corresponding to nuclei image analysis for *soma analysis,*
    j) parameters from d) to f) corresponding to colocalizing image analysis for *colocalization of segments*.

Although *soma analysis* is listed as a separate analysis requiring the addition of nuclei image, neural bodies could be analysed by creating the classifier (for projections, somas and background), and analysing soma class with just *area, number and intensity* option.

2.3. The input parameters

2.3.1. Image folder

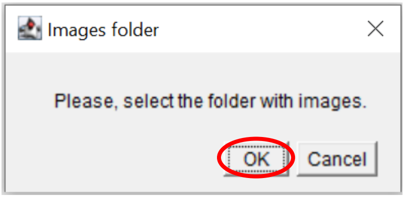

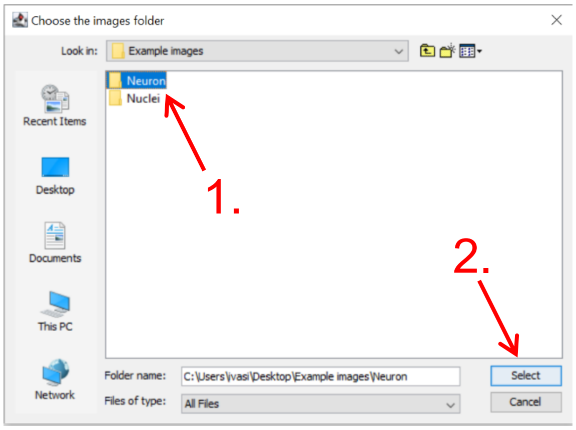

- When this message appears, press “OK” and in the following window select where you placed the images for the analysis. In this folder, only images should be present and all the images that are in the folder will be analyzed by the macro.

2.3.2. Image type, setting scale, cropping and morphological analysis

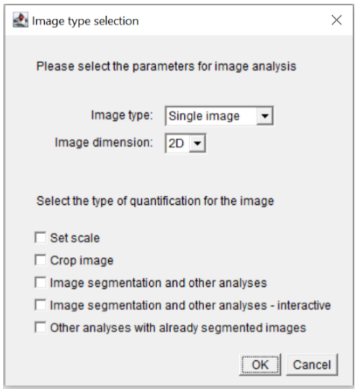

- **Image type** – single image is defined as image with only one channel (see Example images folder), while Channel image is an image containing multiple channels (for example images taken with confocal microscope that have green, blue, red channels – see Example images folder).
- **Image dimensions** – 2D are defined with only one image slice, while 3D symbolizes images with multiple slices (for example z-stack or magnetic resonance angiography).
- **Set scale** option offers the user the ability to set scale on the analysed images.
- **Crop image** allows user to analyze only one part of the images. The selected part will be analysed on all selected images, channels and classes.
- **Image segmentation and other analyses** could be chosen with or without the interactive part. The interactive part is recommended for the first-time users when further input parameters from d) to g) or j) are unknown to the user.
- **Other analyses with already segmented images** option includes the same analysis options but without image segmentation. This option requires the user to have images segmented with other method saved in different folder. Those images are further used for morphological analysis.

2.3.2.1. *Channel information*

It needs to be defined only if in the previous step you selected "Channel image".

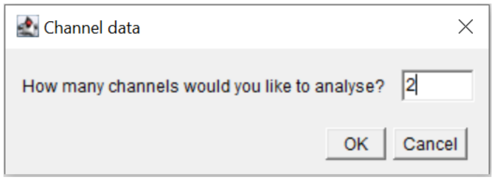

- **How man channels would you like to analyse? –** the total count of channels of the image will be analysed with Lusca.

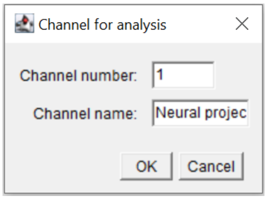

- The next step is repetitive, and the number of repetitions depends on the input data from the previous step.
- **Channel number –** the number of the channels that will be analysed,
- **Channel name –** the name of the channel used for naming the "Results" folder where all data from the analysis will be saved.

2.3.2.2. *Set scale*

It needs to be defined only if in the previous step you selected "Set scale".

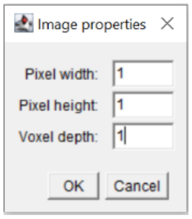

- **Pixel width –** how many pixels can fit in one measured unite looking at x axis (e.g. micrometer, millimeter, inch)
- **Pixel height –** how many pixels can fit in one measured unite looking at y axis (e.g. micrometer, millimeter, inch)
- **Voxel depth –** how many pixels can fit in one measured unite looking at z axis (e.g. micrometer, millimeter, inch)

2.3.2.3. *Crop image*

It needs to be defined only if in the previous step you selected "Crop image".

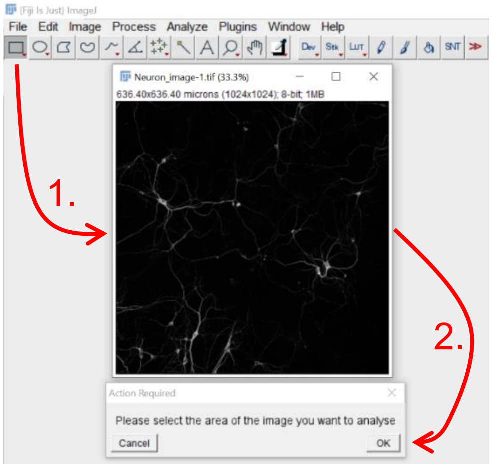

- Crop image option leads the use to the selection of the area with rectangle selection that needs to be analysed. However, the user can choose any other type of selection specific to its needs.
- After the selection, the user presses "OK".

2.3.3. Classifier(s) folder

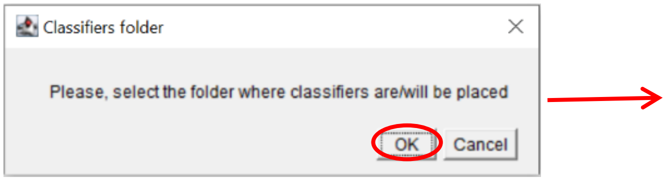

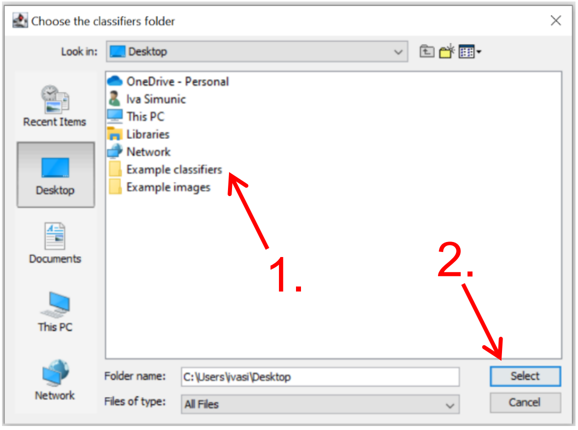

- When the message “Please, select the classifier folder.” appears, press “OK” and in the following window select where image segmentation classifier will be/is placed – depending on whether the input parameters are already made and known.

2.3.4. Analysis for the first batch of images – Interactive part of the macro

It is executed only if you select “Image segmentation and other analyses – Interactive” – we do recommend selecting this if you are an unexperienced user.

2.3.4.1. Classifier formation and the number of classes

- Lusca for image segmentation implements machine learning algorithm, Trainable Weka Segmentation, which requires formation and training of a classifier.
- Before starting with classifier training, it is important to select adequate training features, which depend on the object’s characteristics, in **“Settings”**. Increasing number of the features increases time needed to perform segmentation.
- Depending on the image, user chooses on how many different segments the image will be divided. Those segments are called classes. Classes can be added by **“Create new class”** command, while in **“Settings”** renaming can be done.

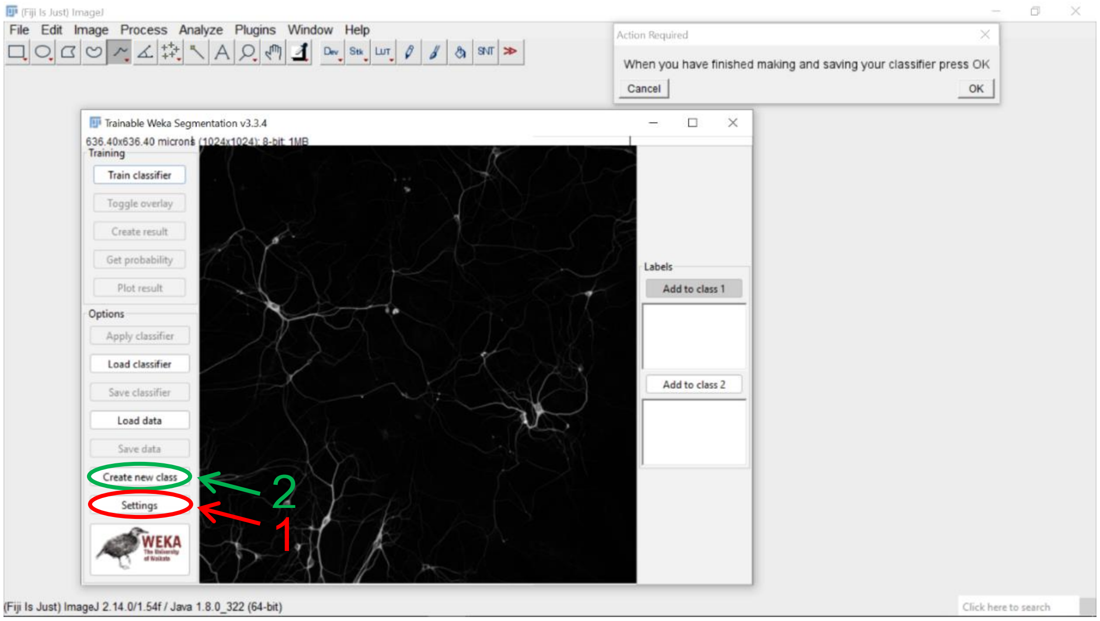

- To start training classifier, to each class labels need to be added. Labels are the example area of the object which the user wants to segmentate. **These areas are selected with five FIJI selection tools**. After selecting the area, user adds the selection into the corresponding class by **"Add to …"**.
- When all the classes have corresponding label/labels, training classifier starts when clicking on the **"Train classifier"**. After training is finished, the overlay on the image will be shown to direct the user which areas are misplaced. Further, the user by selecting them and adding them in the appropriate class, corrects and trains the classifier until it becomes precise.
- When classifier precision is met, it is being saved with **"Save classifier"** into the folder selected in the previous step.
- When the process is finished, the user presses **"OK"** to start the next step.

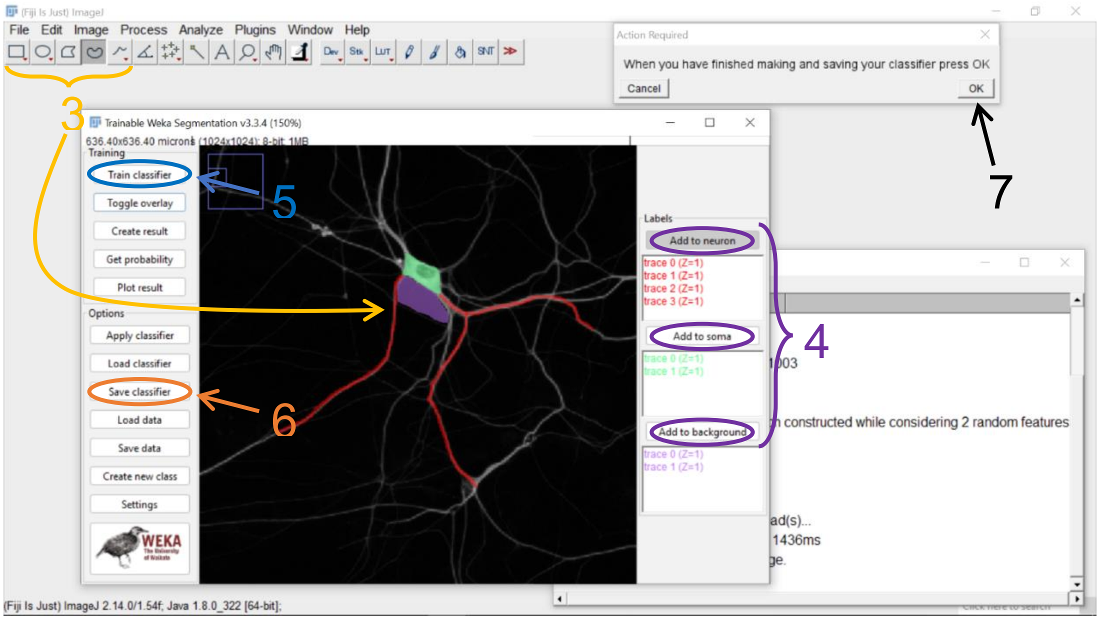

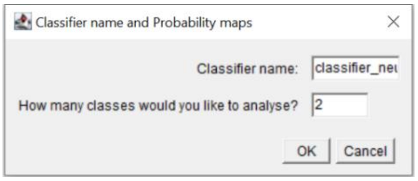

- **Classifier name** – the name of the saved classifier that will be used for image segmentation in Trainable Weka Segmentation (with .model extension),
- **How many classes would you like to analyse?** – total number of classes from Trainable Weka Segmentation that will be analysed. Further input data (class number and name, intensity, area/volume, circularity thresholds, and type of morphological analysis) will repeat as many times as entered here.

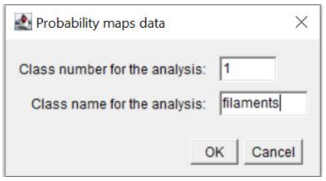

- **Class number for the analysis** – the number of Probability maps channel which corresponds to the class number in Trainable Weka Segmentation that the user would like to analyse,
- **Class name for the analysis** – the name of the class that will be analysed used to make "Results" folder where the images/histograms/detailed result tables macro made during the analysis will be saved.

2.3.4.2. *Intensity threshold*

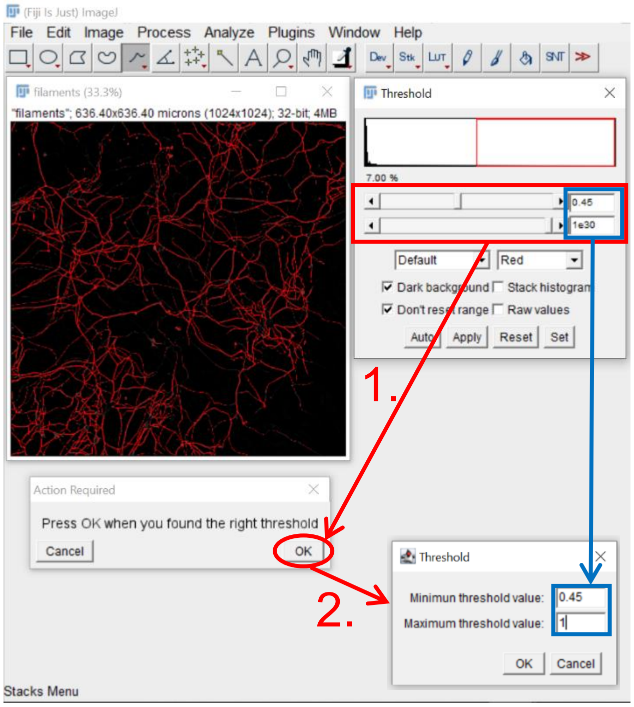

- The result of Trainable Weka Segmentation is Probability maps – a channel image (the number of channels depend on the number of classes) on which whiter pixels correspond to higher probability that they belong to selected class.
- To set the threshold the "Threshold" window is opened. Adjust the two track bars to select the objects you want to analyse and when finished press "OK". Keep in mind that after the threshold you will be able to remove all the small particles with area/volume threshold in the next step.
- **Minimum threshold value** – lower intensity value below which pixels intensity values won't be considered during the analysis (type in the upper track bar value you defined)
- **Maximum threshold value** – upper intensity value above which pixels intensity values won't be considered during the analysis (type in the lower track bar value you defined)

2.3.4.3. *Area/volume and circularity threshold*

- To improve image segmentation and remove excess particles left from the previous step this step is added. To easily assess the settings for input parameters, this step is regulated with "while" loop. As long as the answer to the question below is "No", the loop repeats (**red arrows**), while when answered "Yes" the loop stops and the final values for area/volume are entered (**green arrows**).
- **Minimum particle size** – lower area/volume value below which objects won't be considered during the analysis,
- **Maximum particle size** – upper area/volume value above which objects won't be considered during the analysis,
- **Particle circularity value** – a range from 0.00 to 1.00 (0.00 being not circular object, 1.00 being circular object) in which objects will be analysed,
- **Exclude on the edges** – when marked, objects that are on the edges will not be considered during the analysis

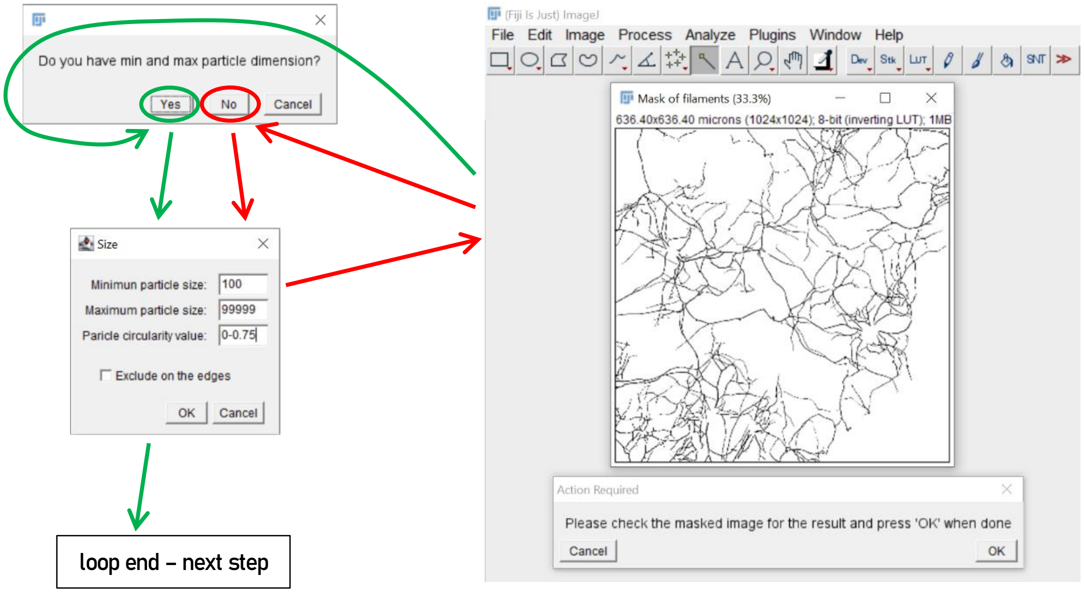

2.3.4.4. *Type of morphological analysis*

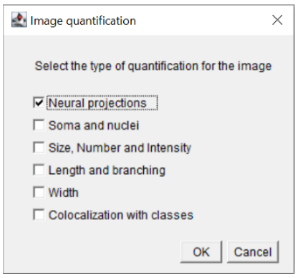

- Types of quantification Lusca calculates are: "Neural projections", "Soma and nuclei", "Size, Number and Intensity", "Length and branching", "Width", and "Colocalization with classes". Detailed list of morphological parameters for each quantification type is given in 2.4. section. User selects aforementioned quantification type(s), depending on wanted result for object morphology.

2.3.4.5. *Optional parameters interactive – neural projections and width*

- To easily regulate the settings for width input parameters, this step is regulated with "while" loop. As long as the answer to the question below is "No", the loop repeats (**red arrows**), while when answered "Yes" the loop stops and the final values for histogram are entered (**green arrows**).
- **Number of bins** – the number used for creating the histogram. This number represents in how many parts the data will be grouped,
- **Minimum histogram number** – the lowest value of data that histogram could obtain,
- **Maximum histogram number** – the highest value of data that histogram could obtain.

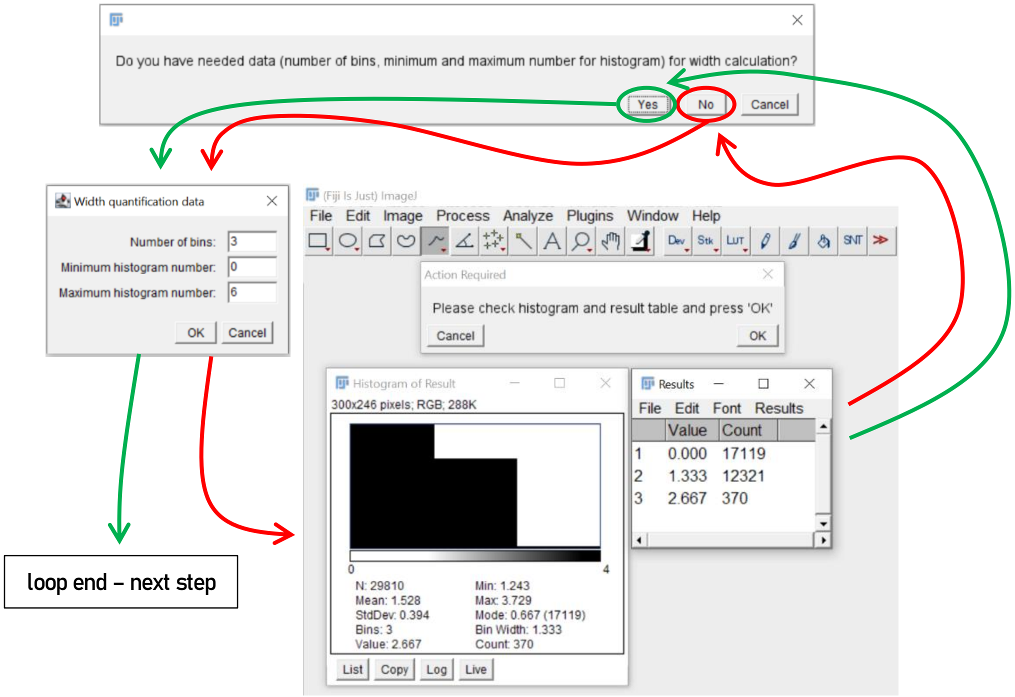

2.3.4.6. *Optional parameters interactive – soma and nuclei analysis*

- The next steps are shown in brief since they are similar to the obligatory steps shown above.
- For single image analysis user select the folder where nuclei images are placed, while for channel images user selects the number of channel with nuclei.

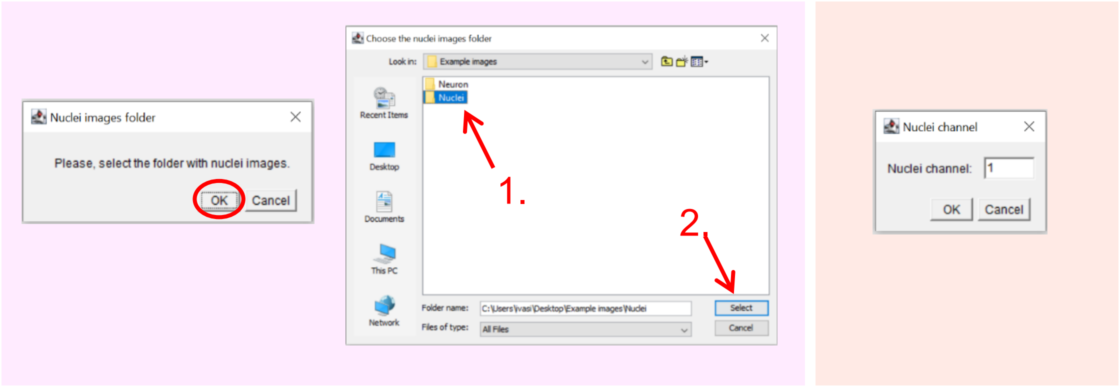

- The formation of the classifier includes the first class as nuclei and the second class as background. The whole process is similar as described in chapter 2.3.4.1. The following steps include naming of the classifier, and setting intensity, area/volume, and circularity thresholds, like the obligatory steps shown above. The option exclude on the edges will be applied to nuclei only if the user selected it on the soma image as well.

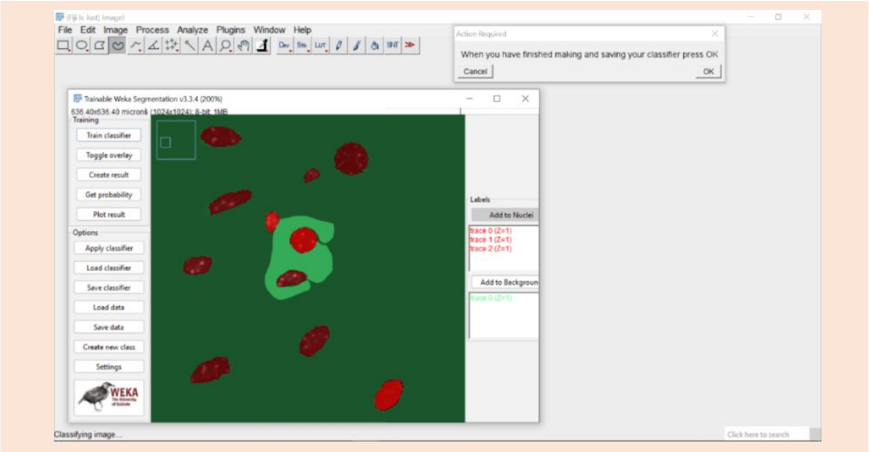

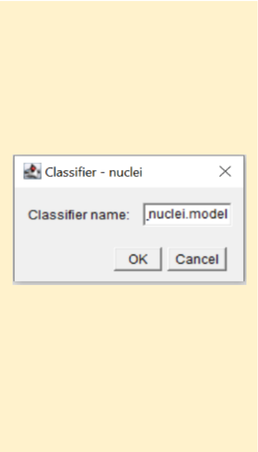

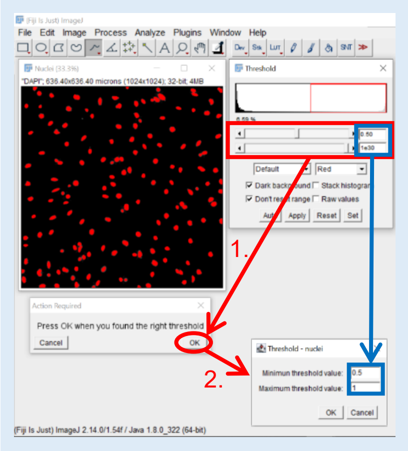

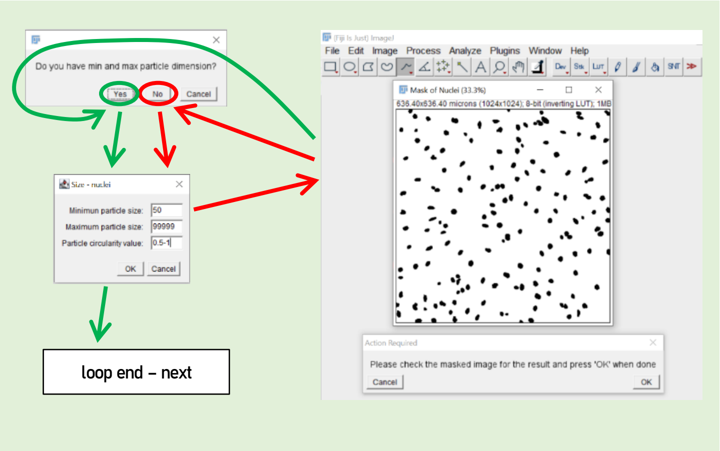

2.3.4.7. *Optional parameters interactive – colocalization analysis*

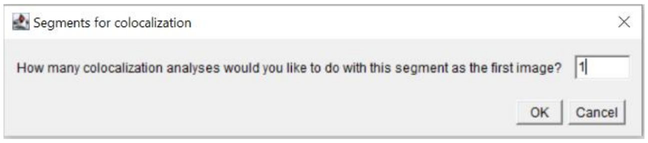

- **How many colocalization analysis would you like to do with this segment as the first image?** – the total number of the colocalization analysis with the image to which parameters have been given.

- For single image analysis user select the folder where second colocalization images are placed, while for channel images user selects the number of channel with second colocalization image.

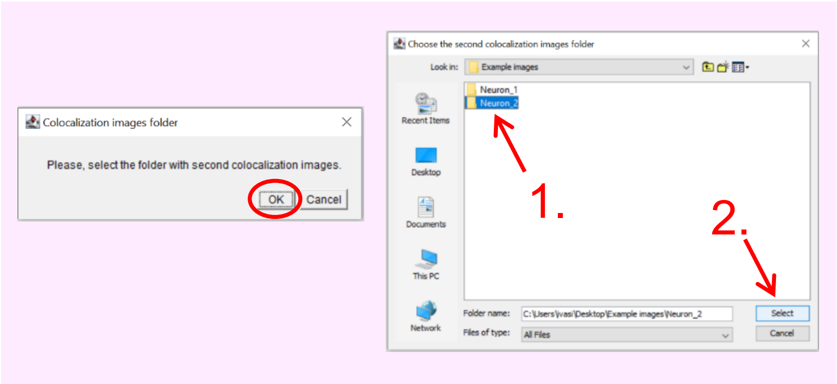

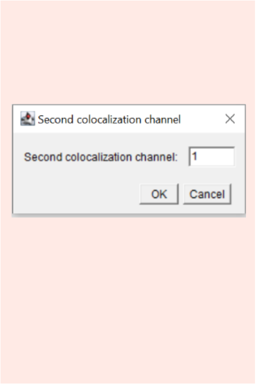

- Further, wizard leads the user to formation of and saving the classifier, naming classifier, and selecting the number of the class with the second objects for colocalization, setting the intensity threshold, along with minimum, maximum and circularity size of the second objects for colocalization. In this step the option exclude on the edges will be applied only if the user selected it on the image with the first objects for colocalization as well.

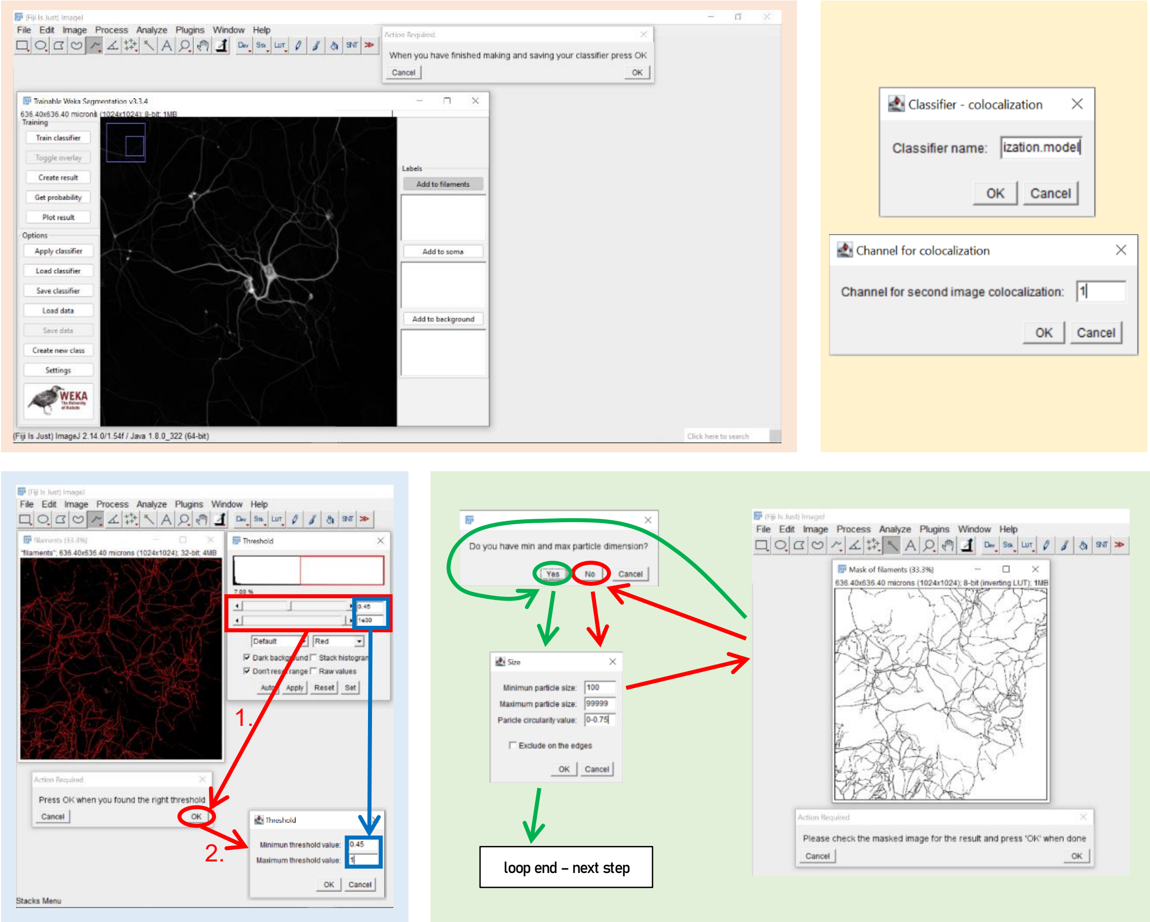

2.3.5. Analysis for the other batches of images – only the input of the parameters

It is executed only if you select "Image segmentation and other analyses".

2.3.5.1. Classifier formation and the number of classes

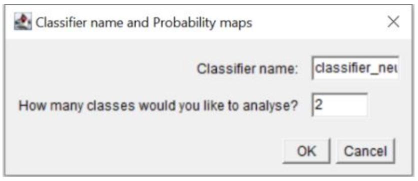

- **Classifier name** – the name of the saved classifier that will be used for image segmentation in Trainable Weka Segmentation (with .model extension),
- **Classes (image segments) count for the analysis** – total number of classes from Trainable Weka Segmentation that will be analysed. Further input data (class number and name, intensity, area/volume, circularity thresholds, and type of morphological analysis) will repeat as many times as entered here.

2.3.5.2. Intensity, area/volume, circularity thresholds and type of morphological analysis

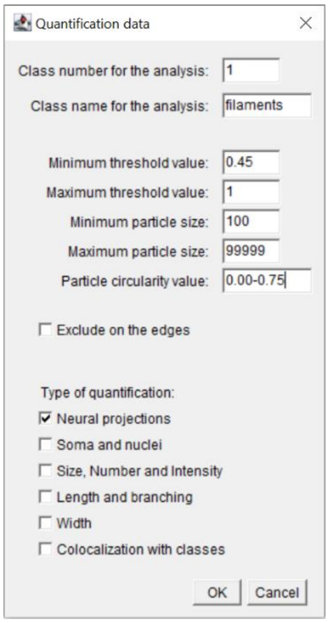

- **Class number for the analysis** – the number of Probability maps channel which corresponds to the class number in Trainable Weka Segmentation that the user would like to analyse,
- **Class name for the analysis** – the name of the class that will be analysed used to make "Results" folder where the images/histograms/detailed result tables macro made during the analysis will be saved.
- **Minimum threshold value** – lower intensity value below which pixels intensity values won't be considered during the analysis,
- **Maximum threshold value** – upper intensity value above which pixels intensity values won't be considered during the analysis,
- **Minimum particle size** – lower area/volume value below which objects won't be considered during the analysis,
- **Maximum particle size** – upper area/volume value above which objects won't be considered during the analysis,
- **Particle circularity value** – a range from 0.00 to 1.00 (0.00 not circular object, 1.00 circular object) in which objects will be analysed,
- **Exclude on the edges** – when marked, objects that are on the edges will not be considered during the analysis,
- **Type of quantification** – type of morphological results, listed detailed in 2.4. section, that will be saved to the "Results" table in the end of the analysis. User selects quantification type(s), depending on wanted result for object morphology.

2.3.5.3. Optional parameters – neural projections and width

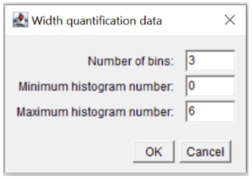

- **Number of bins** – the number used for creating the histogram. This number represents in how many parts the data will be grouped,
- **Minimum histogram number** – the lowest value of data that histogram could obtain,
- **Maximum histogram number** – the highest value of data that histogram could obtain.

2.3.5.4. Optional parameters – soma and nuclei analysis

- For single image analysis user select the folder where nuclei images are placed, while for channel images user selects the number of channel with nuclei.

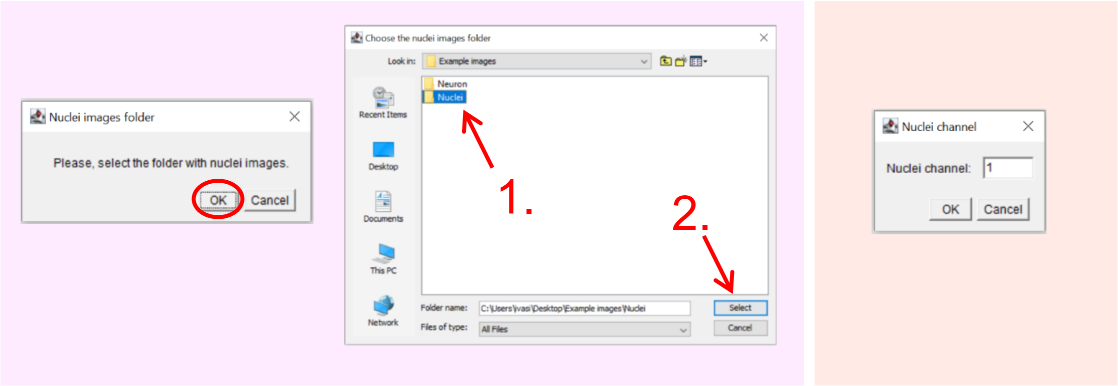

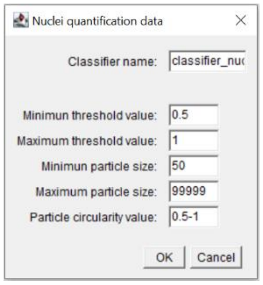

- The last step is entering classifier name for nuclei segmentation (nuclei class always must be the first), minimum and maximum threshold for Probability maps of nuclei, along with minimum, maximum and circularity size for nuclei. In this step option exclude on the edges will be applied to nuclei only if the user selected it on the soma image as well.

2.3.5.5. Optional parameters interactive – colocalization analysis

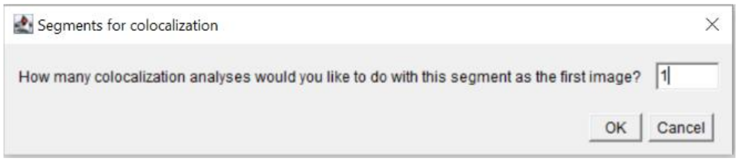

- **How many colocalization analysis would you like to do with this segment as the first image?** – the total number of the colocalization analysis with the image to which parameters have been given.

- For single image analysis user select the folder where second colocalization images are placed, while for channel images user selects the number of channel with second colocalization image.

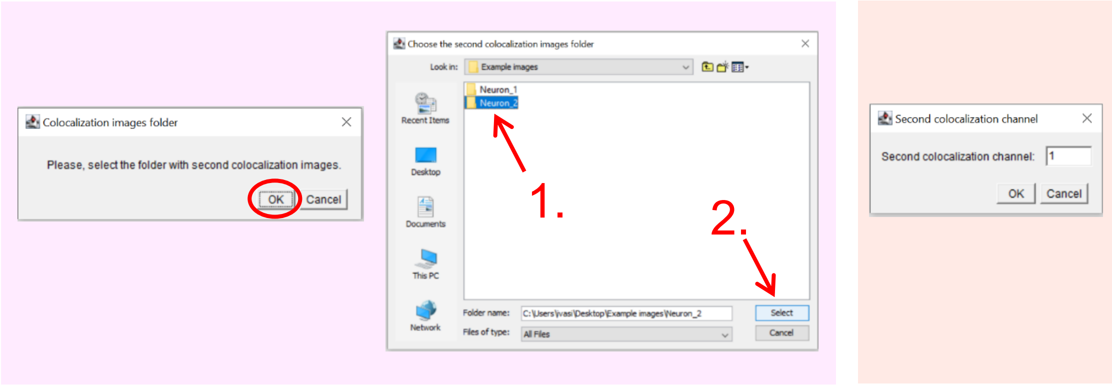

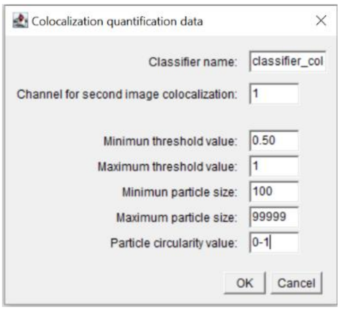

- The last step is entering classifier name for second colocalization image segmentation and the number of a channel with the second objects for colocalization analysis (channel for the second image colocalization), minimum and maximum threshold, along with minimum, maximum and circularity size of the second objects for colocalization. In this step the option exclude on the edges will be applied only if the user selected it on the image with the first objects for colocalization as well.

2.3.6. Image analysis without image segmentation

It is executed only if you select "Other analyses with already segmented images".

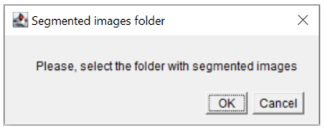

- **Please, select the folder with segmented images –** user selects the folder with images, that correspond to the images in the folder with raw data images, which were segmented with other method.

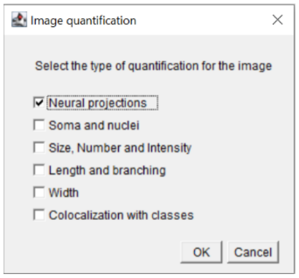

- **Type of quantification –** type of morphological results, listed detailed in 2.4. section, that will be saved to the "Results" table in the end of the analysis. User selects quantification type(s), depending on wanted result for object morphology.

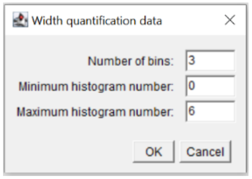

- **Number of bins –** the number used for creating the histogram. This number represents in how many parts the data will be grouped,
- **Minimum histogram number** – the lowest value of data that histogram could obtain,
- **Maximum histogram number** – the highest value of data that histogram could obtain.

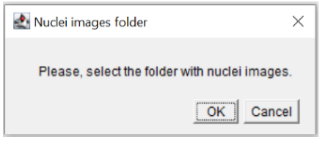

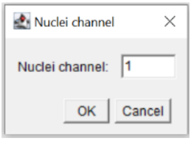

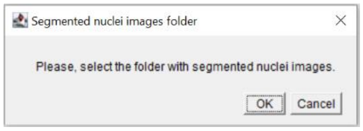

- For single image analysis user select the folder where nuclei images are placed, while for channel images user selects the number of channel with nuclei.
- **Please, select the folder with segmented nuclei images –** user selects the folder with nuclei images, that correspond to the images in the folder/channel with raw data nuclei images, which were segmented with other method.

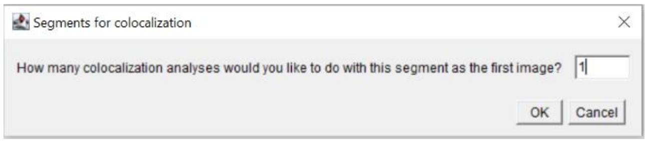

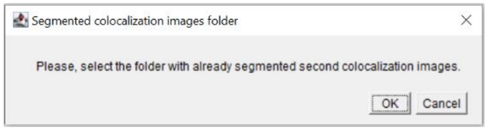

- **How many colocalization analysis would you like to do with this segment as the first image?** – the total number of the colocalization analysis with the image to which parameters have been given.
- **Please, select the folder with segmented second colocalization images** – user selects the folder with second images used for colocalization measurements which were segmented with other method.

2.4. The morphological parameter results

Neural projections	Neural bodies	Number, area/volume and intensity	Length and branching	Width	Colocalization with classes
Count	Soma Count	Count	Total length	Mean width	Segmented M1 and M2
Total Area/Volume	Soma Total Area/Volume	Total Area/Volume	Max branch length	Max width	
Total Surface (3D only)	Soma Total Surface (3D only)	Total Surface (3D only)	Mean branch length	Min width	
Average Area/Volume	Soma Average Area/Volume	Average Area/Volume	Number of branches	Histogram with corresponding table	
Mean Intensity	Soma Mean Intensity	Mean Intensity	Number of junctions		
Circularity/Sphericity	Soma Circularity/Sphericity	Circularity/Sphericity	Number of endpoints		
Total length	Nuclei Count				
Max branch length	Nuclei Total Area/Volume				
Mean branch length	Nuclei Total Surface (3D only)				
Number of branches	Nuclei Average Area/Volume				
Number of junctions	Nuclei Mean Intensity				
Number of endpoints	Nuclei Circularity/Sphericity				
Mean width					
Max width					
Min width					
Histogram with corresponding table					

2.5. End of the analysis

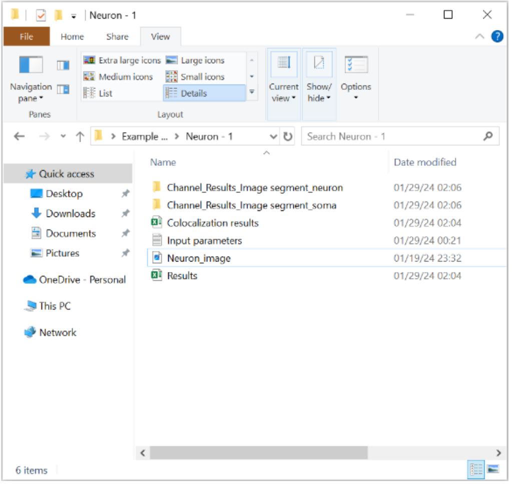

- At the end of the analysis all the results are summed up in the table "Results", input parameters can be found in "Input parameters" text file, while all other images/histogram/tables are saved in the results folder with corresponding name for the Channel and Probability maps name.
- Colocalization results are summed up in the table named "Colocalization results".

3. Troubleshooting and support

All results tables can be opened in Excel. However, it might happen depending on the Excel settings that data are not clearly displayed into columns, but they are separated with the comma in one cell per row. This could be easily formatted by selecting the cells with unsplit data, go to "Data" and "Text to Columns". Select "Delimited" option and "Next". Select "Comma" and "Finish".

For all questions, suggestions, bug reports, and problems related to the Lusca, please feel free to contact: iva.simunic@mef.hr or iva.simunic25@gmail.com.

4. Citation

If you use Lusca for your research, please be so kind to cite our work: "Lusca – FIJI (ImageJ) based tool for automated morphological analysis of cellular and subcellular structures".

5. License

This program is free software; you can redistribute it and/or modify it. This program is supplied in the hope that it will be useful. It is provided without any warranty, not even the implicit warranty of merchantability or fitness for a particular purpose.
